# COPI Complex Is a Regulator of Lipid Homeostasis

**DOI:** 10.1371/journal.pbio.0060292

**Published:** 2008-11-25

**Authors:** Mathias Beller, Carole Sztalryd, Noel Southall, Ming Bell, Herbert Jäckle, Douglas S Auld, Brian Oliver

**Affiliations:** 1 Laboratory of Cellular and Developmental Biology, National Institute of Diabetes and Digestive and Kidney Diseases, National Institutes of Health, Bethesda, Maryland, United States of America; 2 Max-Planck-Institut für biophysikalische Chemie, Abteilung für Molekulare Entwicklungsbiologie, Göttingen, Germany; 3 GRECC/Geriatrics, Veterans Affairs Medical Center, Department of Medicine, School of Medicine, University of Maryland, Baltimore, Maryland, United States of America; 4 NIH Chemical Genomics Center, National Institutes of Health, Bethesda, Maryland, United States of America; CNRS-Universite de Nice Parc Valrose, France

## Abstract

Lipid droplets are ubiquitous triglyceride and sterol ester storage organelles required for energy storage homeostasis and biosynthesis. Although little is known about lipid droplet formation and regulation, it is clear that members of the PAT (perilipin, adipocyte differentiation related protein, tail interacting protein of 47 kDa) protein family coat the droplet surface and mediate interactions with lipases that remobilize the stored lipids. We identified key *Drosophila* candidate genes for lipid droplet regulation by RNA interference (RNAi) screening with an image segmentation-based optical read-out system, and show that these regulatory functions are conserved in the mouse. Those include the vesicle-mediated Coat Protein Complex I (COPI) transport complex, which is required for limiting lipid storage. We found that COPI components regulate the PAT protein composition at the lipid droplet surface, and promote the association of adipocyte triglyceride lipase (ATGL) with the lipid droplet surface to mediate lipolysis. Two compounds known to inhibit COPI function, Exo1 and Brefeldin A, phenocopy COPI knockdowns. Furthermore, RNAi inhibition of ATGL and simultaneous drug treatment indicate that COPI and ATGL function in the same pathway. These data indicate that the COPI complex is an evolutionarily conserved regulator of lipid homeostasis, and highlight an interaction between vesicle transport systems and lipid droplets.

## Author Summary

Fat cells, and cells in general, convert fatty acids into triglycerides that are stored in droplets for future use. Despite the enormous importance of lipid droplets in obesity and other disease processes, we know very little about how lipid reserves in droplets are formed and how those reserves are drawn down. We have used the model fruit fly *Drosophila* to identify candidate regulators of lipid storage and utilization, and have shown that many of these candidates have functions that are conserved in mammals. We focused our attention on a vesicle-trafficking pathway that we show is required for the modulation of the types of regulatory and enzymatic proteins found on the lipid droplet surface. Interfering with the function of this trafficking system with either RNA interference or small-molecule compounds alters lipid storage. The understanding of this new pathway, as well as the specific reagents we used, may ultimately lead to new therapeutics.

## Abstract 3

A specific vesicle-trafficking machine is shown to be required for cells to use stored lipid, in both*Drosophila* and mammalian cells.

## Introduction

Lipid homeostasis is critical in health and disease, but remains poorly understood (for review see [1]). Non-esterified free fatty acid (NEFA) is used for energy generation in beta-oxidation, membrane phospholipid synthesis, signaling, and in regulation of transcription factors such as the peroxisome proliferator-activated receptors (PPARs). Essentially all cells take up excess NEFA and convert it to energy-rich neutral lipids in the form of triglycerides (TG). TG is packaged into specialized organelles called lipid droplets. NEFA is regenerated from lipid droplet stores to meet metabolic and energy needs, and lipid droplets protect cells against lipotoxicity by sequestering excess NEFA. Lipid droplets are the main energy storage organelles and are thus central to our understanding of energy homeostasis. Despite their importance, we know very little about the ontogeny and regulation of these organelles.

Lipid droplets are believed to form in the ER membrane by incorporating a growing TG core between the leaflets of the bilayer, and ultimately are released surrounded by a phospholipid monolayer. Cytosolic lipid droplets possess a protein coat and grow by synthesis of TG at the lipid droplet surface [2] and by fusion with other lipid droplets [3]. Formation of nascent droplets and aggregation of existing droplets is likely to require a dynamic exchange of lipids and proteins from and to the droplet. Indeed, the range of proteins identified in lipid droplet proteomic studies suggests extensive trafficking between lipid droplets and other cellular compartments, including the endoplasmic reticulum (ER) [4–6]. Additionally, lipid droplet-associated proteins translocate between the cytosol and lipid droplets [7]. For example, tail interacting protein of 47 kDa (TIP47) associates with small, putative nascent, lipid droplets [8–10], but is not found on larger droplets, which are coated by other members of the perilipin, adipocyte differentiation related protein (ADRP), TIP47 (PAT) protein family. Intriguingly, TIP47 mediates mannose 6-phosphate receptor trafficking between the lysosome and Golgi [11], raising the possibility that trafficking is involved in lipid droplet ontogeny or fate. However, unlike the well-studied Golgi trafficking system, the routes to and from the lipid droplet are unknown.

Once lipid droplets are formed, stored TG is mobilized in a regulated manner. Triglyceride, diglyceride (DG), and monoglyceride lipases convert TG back into NEFA. Most of our knowledge concerning lipolysis is based on extensively studied adipocytes in which at least two lipolytic enzymes have been identified: adipocyte triglyceride lipase (ATGL) [12–14] and hormone sensitive lipase (HSL) [15]. Due to the hydrophobic properties of the lipid droplet TG core, lipases are likely to act at the surface of lipid droplets [16], where members of the PAT protein family regulate lipase access to the TG core. Mammalian genomes encode at least five PAT-proteins. Whereas perilipin is the dominant PAT protein in adipocytes, ADRP is the dominant PAT protein in nonadipose tissues in which it is tightly associated with the lipid droplet surface [17]. PAT members appear to have a hierarchical affinity for the lipid droplet surface. In nonmammalian genomes, there are fewer PAT proteins. For example, two PAT proteins termed lipid storage droplet 1 and 2 (LSD-1 and LSD-2) are found in Drosophila melanogaster [10]. The crucial role of PAT proteins is evolutionary conserved as the absence of perilipin in mice [18,19], or LSD-2 in flies [20,21] results in lean animals. Overexpression of LSD-2 results in obese flies [20]. These data indicate the conserved PAT proteins at the lipid droplet surface are important regulators of energy storage.

It seems likely that PAT proteins protect lipid from lipolysis, but the role of PAT proteins may not be limited to passive steric hindrance of lipase access to the TG core, as illustrated by perilipin. Unphosphorylated perilipin protects the lipid droplet from lipase activity. Following stimulation by protein kinase A (PKA), however, phospho-perilipin acts as a docking site for HSL [22,23], which translocates from the cytosol to the droplet surface [24]. Whereas phospho-perilipin promotes massive NEFA release from the droplet, this is not mediated exclusively by HSL, as mice lacking HSL function show a relatively mild phenotype marked by the accumulation of DG, thus demonstrating that HSL acts as a DG lipase in vivo [25]. The TG lipase functioning in HSL null mice is ATGL. In the current view of adipocyte lipolysis, ATGL is responsible for the first step in TG hydrolysis, liberating DG and NEFA, whereas HSL acts as a DG lipase. We know very little about how ATGL is targeted to the lipid droplet.

In contrast to the lean phenotype in animals lacking perilipin (mouse) or LSD-2 (fly), both mice and flies lacking ATGL are obese. In mice, the absence of ATGL results in excessive TG accumulation in liver and muscle [12,14]. Similarly, human patients suffering from neutral lipid storage disease carry mutations resulting in truncated ATGL isoforms [26]. ATGL function is evolutionary conserved, as flies lacking the *Drosophila* ATGL ortholog, Brummer, accumulate copious amounts of body fat [13]. The lipid droplet-associated protein Comparative Gene Identification-58 (CGI-58) acts as an ATGL colipase [27]. Mutations in the *CGI-58* gene result in ectopic fat accumulation in patients suffering from Chanarin Dorfman Syndrome (CDS, [28]), supporting the idea that both ATGL and CGI-58 are required for mobilizing lipid stores in nonadipose tissue. Interestingly, CGI-58 physically interacts with perilipin as demonstrated by both coimmunoprecipitation and fluorescence resonance energy transfer (FRET) studies [22,29,30]. In addition, there are other lipases and probably many more cofactors encoded in the genome. Understanding which ones act at the lipid droplet surface and how their localization is regulated will be important.

*Drosophila* is a powerful model for pathway discovery due to well-developed genetics. Additionally, greater than 60% of the genes associated with human disease have clear orthologs in *Drosophila* [31]. *Drosophila* is highly relevant to lipid droplet study, as lipid droplets in *Drosophila* and mammals are associated with many of the same proteins [4–6,32–35]. Finally, the emerging model of lipid storage and endocrine regulation are similar in humans and *Drosophila* [36], suggesting that *Drosophila* will be a good genetic model for lipid storage and lipid storage diseases in humans. We therefore utilized genome-wide RNA interference (RNAi) screening in *Drosophila* tissue culture cells to identify and characterize novel regulators of lipid storage. We then tested for the function of these regulators in mouse lipid droplet regulation by directed RNAi studies. We identified 318 *Drosophila* genes required to limit lipid storage and 208 *Drosophila* genes required to promote lipid storage. These genes encode known regulators of lipid storage as well as genes not previously associated with lipid storage regulation.

Because the protein composition of the lipid droplet surface is so critical for lipid droplet function, and because very little is known about how lipid droplet decoration is regulated, we focused on the exciting finding that the retrograde vesicle-trafficking machinery, utilizing the Coat Protein Complex I (COPI) and COPI regulators, was required to utilize lipid stores. COPI subunit knockdown by RNAi, as well as COPI inhibition with compounds, resulted in increased lipid storage both in *Drosophila* and mouse tissue culture cells, demonstrating evolutionary conservation of our findings.

COPI and COPII vesicles are essential components of the trafficking machinery cycling between the ER and Golgi (reviewed in, e.g., [37]). COPI vesicles mediate cargo transport from the Golgi back to the ER, including escaped ER-resident proteins. The anterograde counterpart, COPII, mediates transport of proteins and lipids from the ER to the Golgi. Whereas interference with either COPI or COPII complexes disrupts Golgi function [38,39], only COPI was required for lipid droplet utilization, clearly demonstrating that COPI and not general Golgi function is required for TG utilization. Although we certainly do not rule out communication between the Golgi and lipid droplet, we suggest that there is a novel ER/lipid droplet trafficking system using a subset of the ER/Golgi transport machinery.

We found that the basis for lipid overstorage following COPI knockdown was a decreased lipolytic rate. Using our existing knowledge of the PAT family members and lipases in the regulation of lipolysis, we examined changes in protein composition at the lipid droplet surface. Interestingly, we found that interfering with the COPI pathway results in ectopic accumulation of TIP47 at the lipid droplet surface. Furthermore, ATGL at the lipid droplet surface was greatly reduced. Combining the effects of ATGL knockdown and compounds affecting COPI function did not elicit a stronger decrease in lipolysis, indicating that ATGL and COPI are both part of the same lipolytic pathway. Thus, our studies provide a functional link between COPI retrograde trafficking and the proteins at the lipid droplet surface. More generally, these results indicate that *Drosophila* RNAi screening is suited to detect uncharted pathways affecting NEFA regulation and to achieve a deeper understanding of cellular lipid droplet regulation.

## Results

### Genome-Wide RNAi to Identify Regulators of Lipid Storage in *Drosophila*

**Figure 1:**
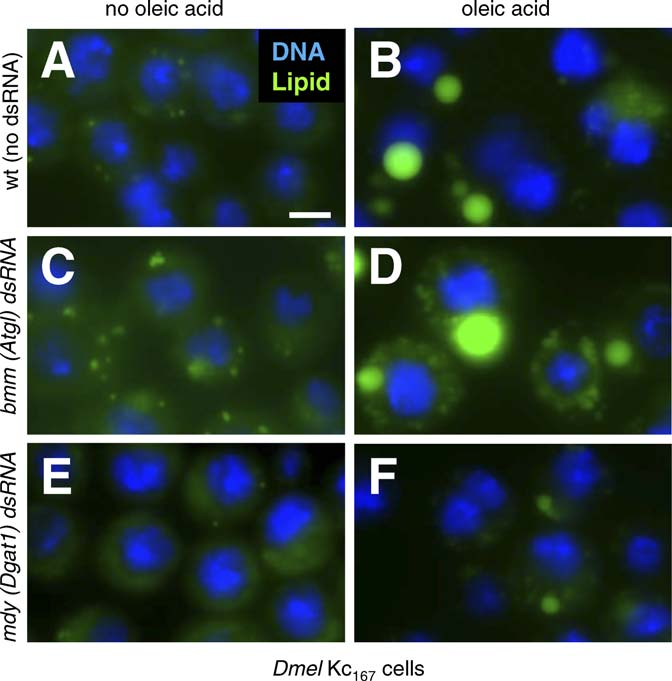
*Drosophila* Cells as a Model of Lipid Storage (A–F) *Drosophila* Kc_167_ cells grown in standard medium (A, C, and E) or medium supplemented with 400 μM oleic acid complexed to 0.4% BSA (B, D, and F). Cells were treated with dsRNA targeting *brummer* (*bmm*; [C and D]) or *midway* (*mdy*; [E and F]). The corresponding mouse genes are *Atgl* and *Dgat-1*. Nuclei are shown in blue (stained with Hoechst 33342), and lipid droplets in green (stained with BODIPY493/503). Scale bar in (A) represents 10 μm.

Lipid droplets are well studied in mammalian cells, but *Drosophila* cells have not been extensively used in lipid droplet studies. Lipid droplets are ubiquitous organelles, and we found that *Drosophila* S2 and SL2 (unpublished data), as well as S3 and Kc_167_ cells (this study) accumulated TG in lipid droplets in the presence of excess NEFA. Kc_167_ cells, for example, stored little lipid when grown on standard media (Figure 1A), whereas in the presence of NEFA (400 μM oleic acid), they readily (within 12 h) accumulated TG packaged in droplets (Figure 1B), which we visualized with the lipid-specific dye BODIPY493/503 [40].

Treatment of *Drosophila* cells with double-stranded RNA (dsRNA) decreases, or “knocks down,” transcript levels for genes sharing the dsRNA sequence, a process known as RNAi [41]. To help determine whether *Drosophila* tissue culture is a good model for lipid droplet function, we used RNAi to target genes encoding known lipid droplet regulators. Flies or mice lacking ATGL store more TG than wild type (“overstorage”) [12–14], whereas those lacking diacylglycerol acyl transferase1 (Dgat1), a key enzyme in TG synthesis [42,43], store less lipid (“understorage”). Knockdown of *bmm*, which encodes *Drosophila* ATGL, increased lipid storage as expected (Figure 1C and 1D). Conversely, treating cells with dsRNA targeting *midway* (*mdy*), which encodes *Drosophila* Dgat1, decreased lipid storage (Figure 1E and 1F). Thus, *Drosophila* cells can be used to analyze gene functions necessary to increase as well as decrease lipid storage.

**Figure 2:**
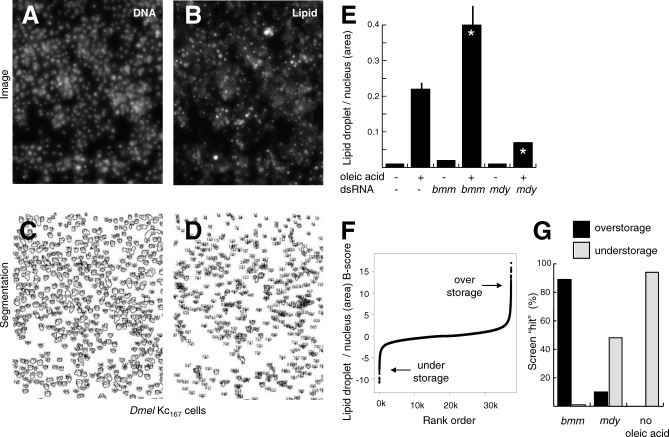
RNAi-Mediated Genome-Wide Screening of *Drosophila* Cells for Lipid Storage Phenotypes (A and B) Regions of original images from the screen of cells stained with DAPI (A) or the lipid droplet-specific dye BODIPY 493/503 (B). (C and D) Image segmentation results of the images shown in (A and B). Nuclei and lipid droplet boundaries are depicted. (E) Sample off-line lipid droplet quantification results by image segmentation. Cells were grown with or without oleic acid and treated with the indicated dsRNAs. Significant differences between dsRNA treated and untreated cells at *p* < 0.05 (two-sided *t*-test) are shown (indicated with an asterisk [*]). Error bars indicate standard deviation. (F) Rank-order plot of the normalized screen results where positive *B*-scores indicate overstorage and negative *B*-scores indicate understorage. (G) Performance of controls incorporated in the on-line screening plates. Black bars represent overstorage (*B*-score > 2.0), and light grey bars represent understorage phenotypes (*B*-score < −1.7).

Although differences in lipid storage are often obvious, we were interested in generating a fully quantitative dataset to support future meta-analysis. To systematically identify and characterize the genes involved in lipid storage, we developed a microscopy-based quantification method based on image segmentation and measurement of nuclear to lipid droplet cross-sectional area (see Figure 2A–2D and Materials and Methods). This technique allowed us to detect lipid storage differences caused by the different feeding conditions and control dsRNA treatments (Figure 2E). We used this imaging method to perform a genome-wide RNAi screen with the well-characterized dsRNA library of the Harvard *Drosophila* RNAi Screening Center (DRSC). This collection covered more than 95% of the predicted *Drosophila* genes [44]. dsRNAs against *bmm* and *mdy* were included in each screening plate as controls. We also included wells with no dsRNA and with or without oleic acid as controls.

As a screening cell line, we used Kc_167_ cells, which showed the best balance of lipid droplet deposition, RNAi susceptibility characteristics, and adhesion during assay development (unpublished data). Following dsRNA treatment of oleic acid-fed cells and image analysis, ratiometric data were normalized within plates and across the entire screening collection using linear models, *B*-score, *Z*-score/median absolute deviation (MAD), and strictly standardized mean difference (SSMD) [45–48], all of which gave similar results. *B*-score normalization [46] across the entire screen marginally out-performed other methods (see Materials and Methods, Table S1). *B*-score results were used for all analyses reported here.

Rank-order analysis of the genome-wide screening results demonstrated that the majority of dsRNAs had no effect on lipid storage. However, two cohorts of dsRNAs resulted in lipid overstorage, as expected for genes required for promoting lipid utilization, or understorage, as expected for genes required for promoting lipid storage (Figure 2F). Thresholds for determining whether a particular dsRNA resulted in a phenotype were selected to balance false negatives and false positives based on the results for *bmm*, *mdy*, and no oleic acid controls. At *B*-scores ≥ 2.0 and ≤ −1.7, greater than 89% of wells treated with dsRNA targeting *bmm* or without oleic acid resulted in the correct overstorage or understorage call, respectively (Figure 2G). Using these cutoffs, we identified 208 candidate genes required for increasing lipid storage (understorage on knockdown, *B*-score ≤ −1.7, Tables S2 and S9) and 318 required for reducing lipid storage or lipid utilization (overstorage on knockdown, *B*-score ≥ 2.0, Tables S3 and S9). These data suggest that about 3% of the *Drosophila* genome is directly or indirectly involved in lipid storage. All data are available in the supplement (Table S4) and at http://lipofly.mpibpc.mpg.de/ and http://flyrnai.org.

**Figure 3:**
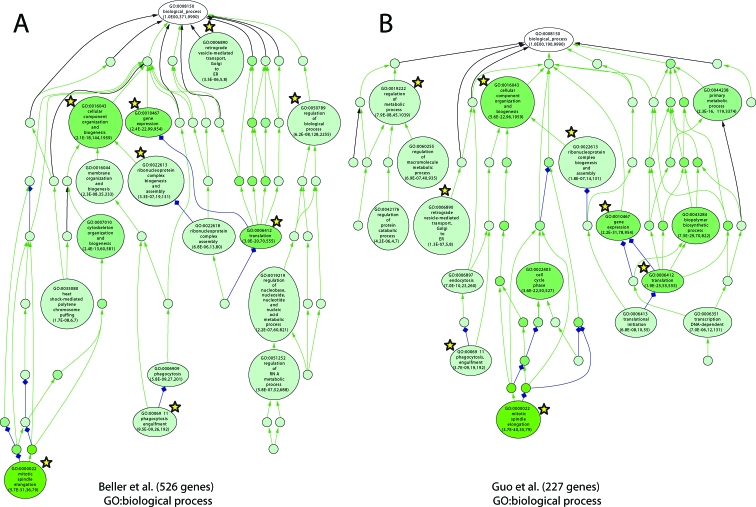
Independent, Genome-Wide RNAi Screens Identify Similar Sets of Genes Regulating Lipid Storage GO-based functional classification (GO: biological process) of genes resulting in lipid storage phenotypes upon targeting by dsRNAs of the present (526 genes) as well as a concurrent study (227 genes; [52]). GO terms were organized into networks using the VLAD software (A and B). Node labels include the associated GO-term identifier and GO-term description as well as the associated *p*-value reflecting enrichment as compared to the whole-genome annotation reference set. The number of genes associated with the respective GO term included in the test set as well as the complete-genome annotation is also shown. A darker node color reflects lower *p*-values. Yellow stars indicate GO-terms shared by the two genome-wide RNAi screen datasets.

**Figure 4:**
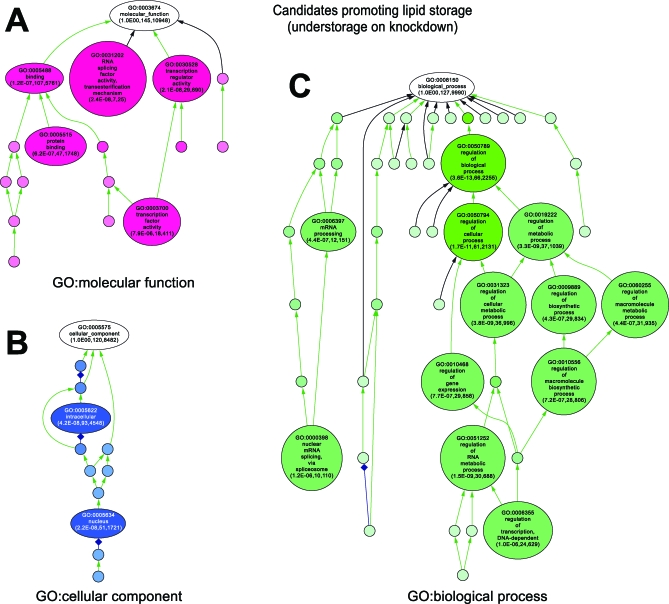
Enriched GO Terms among the Genes Promoting Lipid Storage GO-based functional classification ([A] GO: molecular function; [B] GO: cellular component, and [C] GO: biological process) of genes resulting in lipid understorage phenotypes when targeted by dsRNAs (208 genes). For details, see main text and legend of Figure 3.

**Figure 5:**
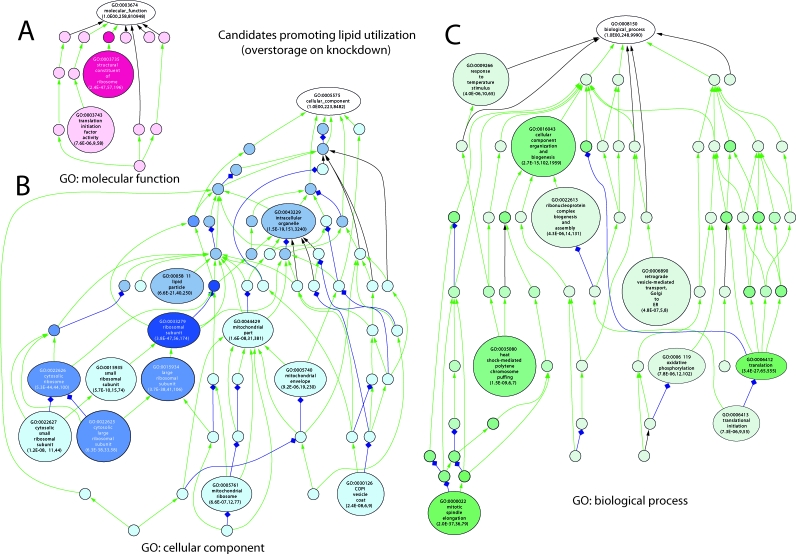
Enriched GO Terms among the Genes Promoting Lipid Utilization GO-based functional classification ([A] GO: molecular function; [B] GO: cellular component, and [C] GO: biological process) of genes resulting in lipid overstorage phenotypes when targeted by dsRNAs (318 genes). For details, see main text and legend of Figure 3.

The most critical test of screen performance is coherence as measured by the identification of multiple genes in a multisubunit complex or a known pathway [49]. Such coherent gene sets are also the best candidates for more detailed analysis. To categorize the dsRNA phenotypes according to molecular networks, we analyzed the identified genes using Gene Ontology (GO) [50] terms with the VLAD tool [51]. This analysis allows for the detection of statistically overrepresented GO terms among a set of genes and projects those enrichments onto the GO-term hierarchy. Genes with a possible function in lipid storage regulation as detected by the RNAi screen were tested against the complete *Drosophila* gene set for enrichment of GO terms associated with biological process, molecular function, and cellular component. Identified, enriched terms were structured in hierarchical networks (Figures 3–5; the results are also tabulated in Table S5). We also took advantage of data from a concurrent lipid storage screen using an independent dsRNA library and *Drosophila* S2 cells [52]. This allows us to develop a robust overview of lipid droplet storage.

Duplication of extensive RNAi screens using different libraries on different cell types provides a cross-validating function that is extremely useful in the analysis of comprehensive datasets. The overlap (25%, 57 genes) between the S2 cell screen (227 genes identified; Table S6) and our genome-wide study on Kc_167_ cells (526 genes identified) was highly significant (*p* < 1e^−14^, Wilcox test). More importantly, the GO term networks were quite similar and suggest that key pathways have been identified (Figure 3). For example, both screens show that interfering with translation factors and ribosomes result in lipid storage defects (GO:0022613, GO:0006412). Additionally, genes resulting in lipid storage defects are enriched for transcriptional regulators in both screens (GO:0010467) and trafficking (GO:0006911, GO:0006890). The only major differences between the screens were that genes involved in pre-mRNA processing were enriched in our Kc_167_ cell screen and genes involved in proteasome function were enriched in the S2 cell screen. However, five genes required for lipid storage in our study (*suppressor of deltex*, *ubiquitin conjugating enzyme 2*, *ubiquitin activating enzyme 1*, *Roc1a*, and *Roc1b*) are involved in ubiquitin-mediated proteolysis at the proteasome [53]. Thus, the screens are largely cross-validating.

Gene knockdowns resulting in understorage have a candidate wild-type function in promoting lipid storage. Whereas we identified gene functions linked to neutral lipid synthesis (Table S5), the most striking enrichments were for regulatory functions within the nucleus (Figure 4). GO terms associated with the nuclear functions transcription or transcript processing were particularly prominent (Figure 4). These data suggest that lipid storage requires a complex regulatory network.

In contrast, the candidate genes required for lipid utilization were enriched for cytoplasmic functions (Figure 5, Table S5). We found that lipid storage increased after treatment with dsRNAs targeting genes encoding lipid droplet-associated proteins (GO:0005811). In addition to GO term analysis, we directly compared the identified candidate lipid storage-modulating genes functions with genes encoding proteins of the recently described, but functionally uncharacterized, lipid droplet-associated mammalian [5,6,32,35] and *Drosophila* [4,33] subproteomes, only some of which have lipid droplet GO terms. These genes were far more likely to result in a lipid overstorage phenotype when subjected to knockdown in *Drosophila* (*p* > 1e^−16^, Wilcox test) than the reference genome-wide dsRNA targets. This suggests that many of the genes revealed by our RNAi experiments encode direct regulators of lipid storage. Gene functions involved in mitochondrial fatty acid beta-oxidation, which utilize NEFA as a substrate, as well as genes involved in protein synthesis, were also enriched. Indeed, knockdown of 12% of the *Drosophila* genes encoding translation-related functions (GO:0006412), including 32% of the genes encoding ribosomal subunits (GO:0033279), resulted in lipid overstorage (Figure 5, Table S5). It is possible that decreased ATP demand for protein synthesis and decreased ATP generation in mitochondria simply decrease the need for energy in the cells, resulting in increased lipid storage. Mitochondrial uncoupling and beta-oxidation pathways are areas of therapeutic interest for diabetes and other metabolic disorders [54–57].

One of the most striking results was the prevalence of cellular transport functions in general (GO:0006909, GO:0006890; and GO:0000022), and the COPI trafficking pathway mediating Golgi to ER transport in particular, among the genes resulting in a lipid overstorage phenotype on knockdown (Figure 5, Table S5). Nascent lipid droplets are thought to form at the ER and then enlarge and fuse to form larger droplets [8–10]. Thus, our result is somewhat surprising, as we expected that wild-type ER functions might be involved in promoting lipid storage rather than lipid utilization. Similarly, it is known that lipid droplets are transported as cargo on microtubules in *Drosophila* embryos and that such transport is required for fusion of lipid droplets in muscle cells [3,58]. There was a strong enrichment for genes involved in spindle microtubule elongation (Figure 5C) among the genes showing overstorage on knockdown. Again, whereas microtubule involvement in lipid storage is predicted, interfering with microtubule cargo transport might be expected to decrease lipid storage.

**Figure 6:**
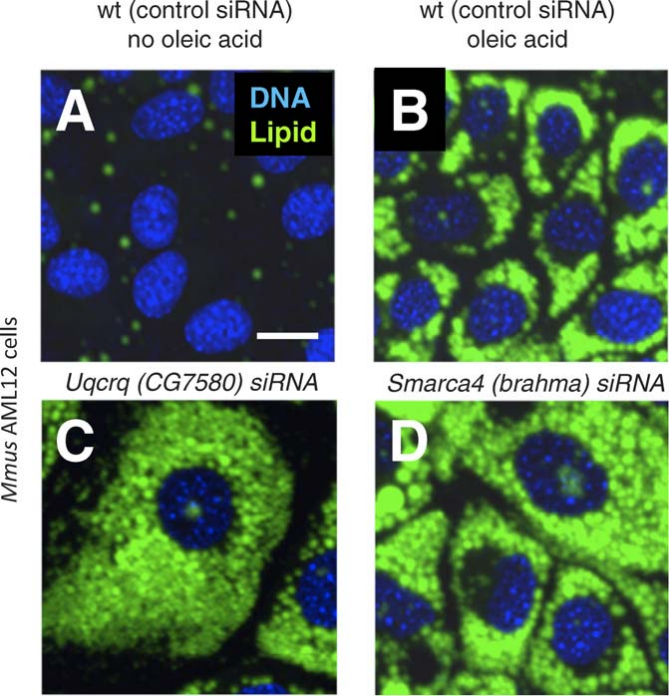
Evolutionary Conserved Lipid Droplet Regulators Mouse AML12 cells store little lipid in the absence of exogenous NEFA (A), whereas addition of NEFA to the growth medium induces lipid droplet deposition (B). Knockdown effects on cellular lipid droplets of the mouse genes encoding Uqcrq (CG7580 in *Drosophila*), an electron transport chain member, and Smarca4 (Brahma in *Drosophila*), a chromatin-associated protein (C and D). Nuclei are shown in blue (stained with Hoechst 33342), lipid droplets in green (stained with BODIPY493/503). Scale bar in (A) represents 50 μm.

To validate a “gold set” of genes ready for extended follow-up, we selected genes for additional *Drosophila* treatments using original and secondary dsRNAs. At least two different nonoverlapping dsRNAs in our screen or in the Guo et al. screen [52] resulted in confirmed understorage or overstorage phenotypes for a subset of candidate genes (Table S7). Additionally, mouse orthologs of 127 *Drosophila* genes selected on the basis of lipid storage phenotypes in Kc_167_ cells (including orthologs of 54 genes that failed to pass our cutoff) were knocked down in two mouse cell lines using short interfering RNAs (siRNAs). We used a mouse fibroblast cell line (3T3-L1), in which lipid droplets have been extensively characterized, and a liver cell line, AML12, which was previously used as a model of ectopic fat deposition [59]. Retesting in mouse cells is a particularly stringent validation of the *Drosophila* dsRNA data as it simultaneously provides information about evolutionary conservation as well as obviating concerns about spurious off-target effects [49,60,61]. The 33 genes resulting in lipid storage defects when knocked down in both *Drosophila* and in mouse cells validate the involvement of many of the biological processes implicated by the primary screen (Table S7). For example, knockdown of the *Ubiquinol cytochrome c reductase complex III subunit VII* gene (*Uqcrq*; ortholog of the *Drosophila CG7580* gene), which encodes a component of the mitochondrial respiration chain, results in greatly enlarged AML12 cells storing dramatically more lipid than control cells (Figure 6A–6C). Similarly, knockdown of *Smarca4* (ortholog of the Drosophila brahma gene), which encodes a member of the SWI/SNF chromatin modifying complex [62], results in lipid overstorage (Figure 6D). Knockdowns of COPI complex members resulted in overstorage in *Drosophila* S2 and Kc_167_ cells, and in mouse 3T3-L1 and AML12 cells (Table S7). Although there is much to be gleaned from the screen, we focused our attention on the Golgi to ER trafficking COPI complex.

### COPI Complex Is a Regulator of Lipid Storage

**Figure 7:**
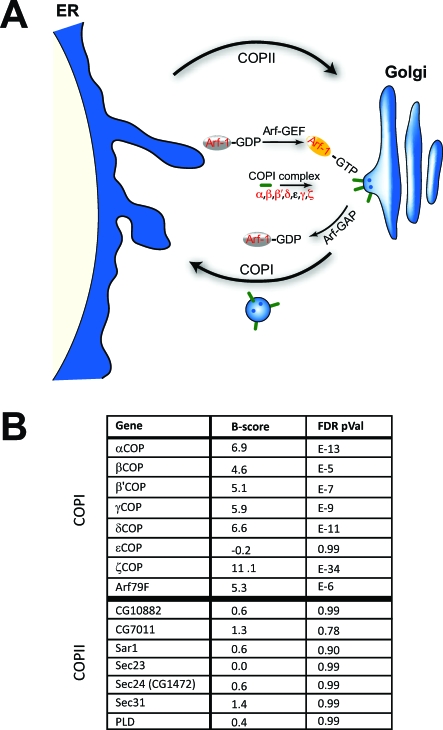
COPI Is a Regulator of Lipid Storage (A) Schematic representation of the COPI trafficking pathway (center) directing traffic from the Golgi (right) to the ER (left). COPII members (not detailed) direct traffic from the ER to the Golgi. Members showing increased lipid storage following knockdown in the *Drosophila* primary screen are highlighted (red). (B) Primary screen results for COPI and COPII components are tabulated. The average *B*-score for the given gene (higher *B*-scores indicate more stored lipid, see Figure 2) as well as the average false discovery rate (FDR)-corrected *p*-value are given.

Overrepresentation of genes encoding ER/Golgi vesicle-associated proteins among the genes showing a lipid overstorage phenotype on knockdown suggests that vesicle trafficking proteins participate in lipid utilization. Most strikingly, six out of the seven genes encoding COPI subunits (Figure 7) that mediate retrograde transport from the Golgi to the ER, showed dramatically increased lipid storage following dsRNA treatment in the genome-wide RNAi screen (*B*-score = 4.6 to 11.1, false discovery rate [FDR]*-*corrected *p* = 1e^−5^ to 1e^−34^). Enrichment for members of such multisubunit complexes in RNAi screens has outstanding predictive value [49]. Our observed enrichment for essentially all the COPI-associated factors among the knockdowns resulting in lipid overstorage, strongly suggests that COPI is required for limiting lipid storage (FDR-corrected *p* < 1e^−6^). In addition, dsRNAs targeting *ADP ribosylation factor at 79F* (*Arf79F*) had the same effect as COPI knockdown. *Arf79F* encodes a small G protein homologous to mammalian Arf1, the key regulator of COPI vesicle formation at the Golgi [63]. Surprisingly, ɛCOP was the only COPI subunit repeatedly failing to produce a lipid storage phenotype following RNAi in both the S2 [52] and our Kc_167_ cell screens. Although this is a negative result, we suggest that this subunit is not involved in lipid storage regulation (see Discussion). Interestingly, none of the seven COPII members required for anterograde transport from the ER to the Golgi [37,38] showed a lipid accumulation phenotype following RNAi (Figure 7B; *B*-score = 0.0 to 1.4, FDR-*p* = 0.99 to 0.78), strongly suggesting that lipid overstorage due to COPI knockdown is not a general consequence of disrupted trafficking between the ER and Golgi.

**Figure 8:**
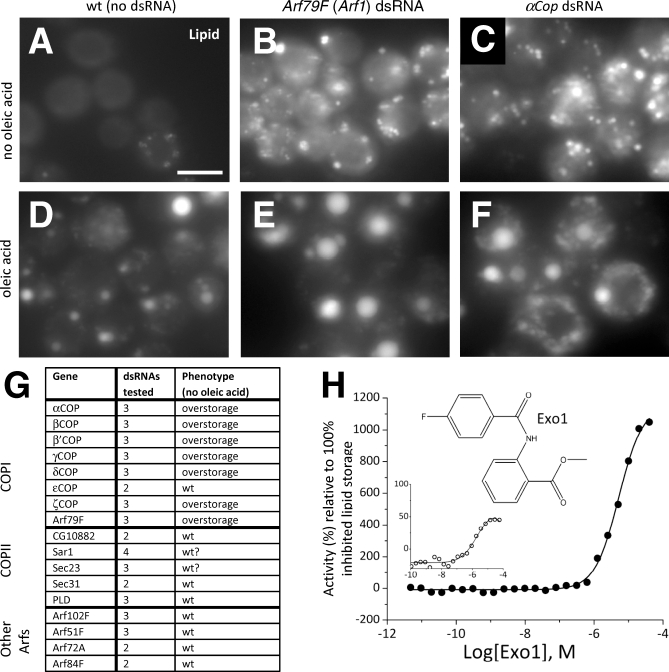
The COPI-Mediated Retrograde Trafficking Pathway Is a Negative Regulator of Lipid Storage (A–F) *Drosophila* cells with or without oleic acid stained with BODIPY493/503 to detect lipid. Control cells not treated with dsRNA (A and D) or cells incubated with dsRNAs targeting *Arf79F* (an Arf1 homolog) (B and E) or α*Cop* (C and F) are shown. (G) All COPI, and several COPII members as well as additional Arfs, were retested using independent dsRNAs and gave similar results. Results and number of dsRNAs present in the primary screen (including oleic acid) and retests are given. (H) Dose response of *Drosophila* S3 cells to Exo1 (structure inset) showing the %-activity derived either from the lipid specific signal (filled circles [•]) or the lipid/cell ratio (open circles [○]). Percent activity refers hereby to the changes of lipid storage relative to Triacsin C treatment, which decreases lipid storage by blocking TG synthesis (this is analogous to the *mdy* controls in RNAi experiments, see Material and Methods). Increased activity indicates increased lipid storage, which increased with concentration. Scale bar in (A) represents 10 μm.

In organisms, cells are exposed to differing NEFA levels due to feeding and fasting. Therefore, to test for the function of the COPI complex in physiological conditions without elevated NEFA, we also performed new RNAi experiments with or without supplementing the media with oleic acid (Figure 8A–8G; additional data not shown). Even in the absence of oleic acid, knockdowns of all the members of the COPI complex that promoted lipid droplet deposition under fed conditions also promoted accumulation without feeding (Figure 8A–8G; additional data not shown). Thus, the lipid storage phenotype was also independent of the nutritional status of the cells.

To further investigate whether the observed lipid storage phenotype after the loss of COPI-subunit function is due to a specific pathway or a more general effect of interference with Golgi and ER integrity, we also tested additional dsRNAs targeting transcripts encoding the COPII-associated proteins CG10882, Sar1, Sec23, Sec31, and PLD (Figure 8G). Furthermore, the *Drosophila* genome encodes five Arf proteins [64], which we also reinvestigated in additional RNAi experiments. Arf79F encodes ARF1, which is required for COPI function, but Arf51F, Arf72A, Arf84F, and Arf102F are not known to be required for COPI-mediated transport [65]. Only Arf79F resulted in a mutant lipid droplet phenotype upon RNAi knockdown (Figure 8G). These experiments demonstrated that the lipid overstorage phenotype is specific to COPI loss of function and raise the possibility that the lipid overstorage phenotype is Golgi independent.

Although multiple dsRNAs verified the phenotypic effect of COPI knockdown, we sought to further validate those results with an independent technique, to rule out effects based on the RNAi treatment, or the prolonged incubation time (4 d) due to the knockdown procedure. Therefore, we also tested pharmacologically for COPI involvement in lipid storage. We treated *Drosophila* S3 cells for 18 h with 24 different concentrations of Exo1, a selective inhibitor of Arf1 activity [66], and determined the dose response (Figure 8H). Lipid droplets were stained with the same dye as for the RNAi experiments. As in the RNAi experiments, we used internal controls, including cells with no oleic acid feeding, cells treated with the compound solvent, and cells treated with Triacsin C, a known inhibitor of TG synthesis [67]. Dose-response curves for Exo1 were determined by enumerating cells that showed an increase in lipid staining (or relative to the enumerated cells based on a cytosolic counterstain; inset in Figure 8H) and expressing this as per cent activity relative to cells incubated in oleic acid and full inhibited by Triacsin C. Thus, increased activity indicates lipid overstorage. Treatment of *Drosophila* S3 cells with Exo1 resulted in a dose-dependent (half maximal effective concentration [EC_50_] = 5 μM) increase in lipid storage that was greater than 10-fold. Thus, multiple dsRNAs targeting COPI and Arf79F mRNAs as well as Exo1, a compound targeting Arf79F (Arf1 in mammals), resulted in the same phenotype. These data strongly indicate that COPI is required to limit lipid storage in droplets in *Drosophila*.

### COPI Complex Is an Evolutionary Conserved Regulator of Lipid Storage

To explore the function of COPI in lipid droplet cell biology in greater detail, we performed additional experiments in the mouse 3T3-L1 and AML12 cells. As positive and negative controls, we used irrelevant “ALLStars negative control” siRNAs, or siRNAs targeting transcripts encoding known lipid droplet regulators, and compared the resulting cellular phenotypes to the results of parallel siRNA treatments targeting transcripts encoding COPI components. As in the *Drosophila* experiments, we required that at least two siRNAs resulted in the same phenotype.

**Figure 9:**
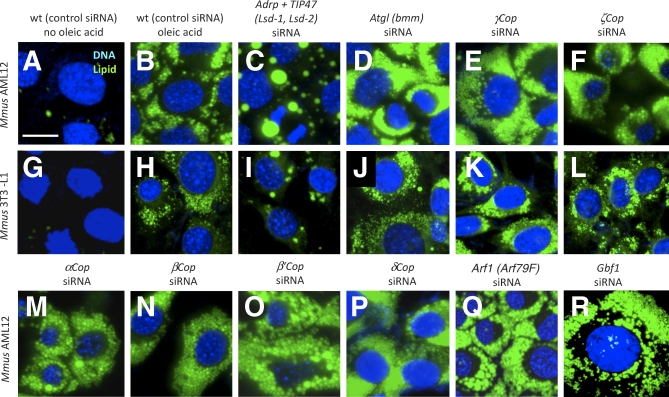
Function of Selected Mouse Orthologs of Genes Showing Lipid Storage Phenotypes in *Drosophila* Cells (A–F and M–R) AML12 or 3T3-L1 cells (G–L), with (B–F and H–R), or without (A and G) oleic acid and stained for nuclei (Hoechst 33342) and lipid (BODIPY493/503). Cells were transfected with ALLStars negative control siRNA (control) or the siRNAs targeting the indicated genes. *Drosophila* homologs are given (parentheses). Scale bar in (A) represents 50 μm.

Like AML12 cells, 3T3-L1 cells also stored little lipid in the absence of exogenous NEFA (Figure 9A and 9G), whereas small, clustered lipid droplets appeared upon addition of oleic acid (Figure 9B and 9H). Depletion of both ADRP and TIP47 by RNAi resulted in fewer and much larger lipid droplets (Figure 9C and 9I [68]) relative to wild type (we used double knockdowns for these controls because single knockdowns resulted in a minimal phenotype [68]). Conversely, knockdown of *Atgl* (*bmm* in *Drosophila*) transcripts resulted in increased lipid storage (Figure 9D and 9J), but no differences in the appearance of the lipid droplets. Targeting the genes encoding α, β, β′, γ, δ, or ζ COPI subunits by siRNAs resulted in increased lipid storage (Figure 9E, 9F, and 9K–9P). As in the *Drosophila* knockdown experiments, ɛCOP knockdown failed to increase lipid storage (unpublished data). We also failed to observe a phenotype following knockdown of either of two genes, *sec24* and *Pld1*, encoding COPII components (unpublished data). Thus, the *Drosophila* and mouse RNAi experiments unambiguously indicate that COPI subunits (with the exception of ɛCOP) have evolutionarily conserved lipid droplet functions.

Both Arf1 and Gbf1, an Arf guanine nucleotide exchange factor (GEF), are required for COPI recruitment from the cytosol to Golgi [69]. We also asked whether Arf1 and any of three pharmacologically related GEFs were required for lipid utilization. The Gbf1, Big1, and Big2 proteins are GEFs inhibited by Brefeldin A (BFA) [70]. BFA treatment and knockdowns of either *Arf1* or *Gbf1* (the latter confirmed at the protein level) resulted in lipid overstorage (Figure 9Q and 9R), whereas we observed no lipid overstorage following knockdown of *Big1* or *Big2* (unpublished data). Thus the COPI complex and critical regulators of COPI translocation are required for lipid utilization.

### Loss of COPI Function Results in Decreased Lipolysis

**Figure 10:**
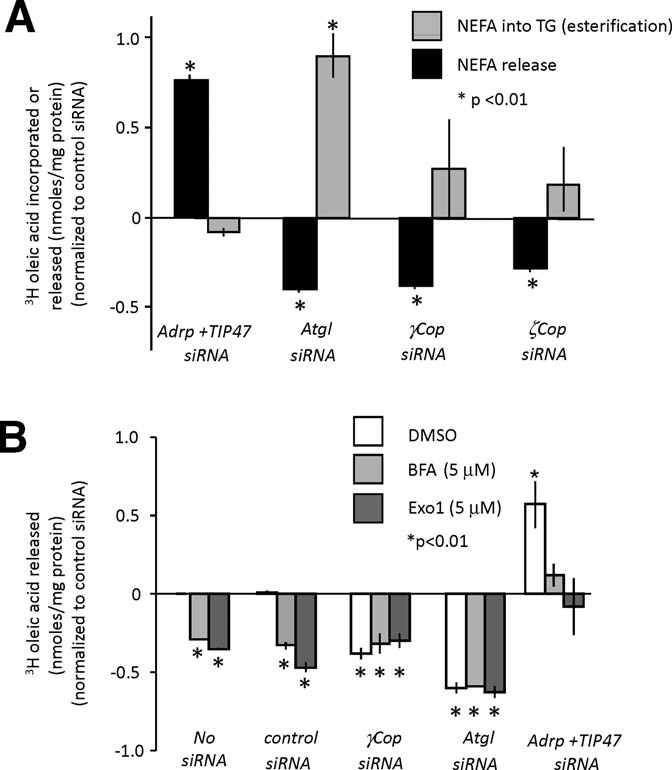
NEFA Incorporation and NEFA Release Measured in AML12 Cells after RNAi-Mediated Gene Knockdown and Drug Treatment (A) Relative activity [(experimental/ALLStars negative control (control)) − 1] in radiolabel assays for TG esterification (nM) (grey bar) and NEFA release (nM) (black) relative to total protein concentration in cells treated with siRNAs targeting the indicated transcripts. Significance at *p* < 0.01, impaired *t*-test, is shown (indicated by an asterisk [*]). Standard error is indicated by the bars. (B) Relative activity [(experimental/ALLStars negative control (control) and DMSO) − 1] in radiolabel assays for NEFA release (nM) relative to total protein concentration in cells treated with siRNAs targeting the indicated transcripts in the presence of DMSO only (open bar), BFA (5 μM) in DMSO (light-grey bar), or Exo1 (5μM) in DMSO (dark-grey bar). Significance at *p* < 0.01, impaired *t*-test, is shown (indicated by an asterisk [*]). Standard error is indicated by the bars.

Lipid overstorage in the absence of COPI could be due to decreased release of NEFA from droplets, or increased synthesis of TG for storage, or both. In order to explore whether COPI is required for one or both of these general functions, we measured both NEFA release and esterification of NEFA into TG in AML12 cells (Figure 10A). As expected, we observed increased release of NEFA from cells treated with control siRNAs targeting *Adrp* and *Tip47* transcripts, which is mediated by increased amounts of lipid droplet-associated ATGL [68]. In contrast, NEFA release decreased when *Atgl* lipase transcripts were targeted as controls. Additionally, we observed increased incorporation of NEFA into TG following *Atgl* knockdown, suggesting that the tremendous increase in TG seen in those cells is due to decreased NEFA release and continued synthesis of TG despite the reduced efflux. The modest increase in incorporation of NEFA into TG following COPI knockdown was insignificant. However, we observed approximately 40% of wild-type NEFA release in cells treated with siRNAs targeting either γCOPI or ζCOPI transcripts—in the same range as after *Atgl* knockdown (Figure 10A). In separate experiments, we also observed decreased NEFA release following *Gbf1* knockdown, but not following *Big1* or *Big2* knockdown (Table S8). These data indicate that COPI is a novel regulator of lipolysis.

We also asked whether short-term pharmacological inhibition of COPI trafficking phenocopies the COPI knockdown phenotype in mouse cells, as we noted in *Drosophila* cells. We used COPI inhibitors Exo1 and BFA [39,66], both of which result in increased lipid storage. Both compounds reduced NEFA release to the same extent as the siRNAs targeting COPI subunit mRNAs (Figure 10B). To dissect the role of COPI in lipolysis, we used a combination of siRNAs targeting different genes in the lipolytic pathway, and Exo1 or BFA treatment, to mimic genetic epistasis experiments (a proven tool for dissecting functional relationships between members of the same or different pathways [71]). Combining siRNA-mediated knockdown of COPI members and BFA or Exo1 treatment did not enhance the decreased lipolysis phenotype (Figure10B), indicating that the observed effects following drug treatment are only COPI mediated. Additionally, these data suggest that there are no serious compound-based side effects vis-a-vis lipid droplets, even for the broad-spectrum inhibitor BFA (also note that other BFA-sensitive GEFs, Big1 and Big2, did not result in a lipid storage phenotype on knockdown). Decreased lipolysis could be due to decreased lipase activity at the lipid droplet. To determine whether that lipase was ATGL, we combined siRNAs targeting ATGL transcripts and BFA or Exo1 drug treatment (Figure 10B). If ATGL were responsible, then ATGL knockdown would have no effect on BFA- or Exo1-treated cells. Indeed, the lipolysis rate was not further decreased, suggesting that COPI-mediated lipolysis effects are mediated by ATGL. This conclusion is further supported by experiments in which we treated cells with siRNAs targeting ADRP and TIP47 transcripts in combination with either BFA or Exo1. In the absence of ADRP and TIP47, more ATGL is found at the lipid droplet surface [68]. We also found that Exo1 or BFA treatment rescues the effect of ADRP and TIP47 knockdown. This, along with the finding that COPI and ATGL are in the same pathway, suggests that COPI is an important positive regulator of ATGL.

### Loss of COPI Function Results in Altered Lipid Droplet Protein Composition

**Figure 11:**
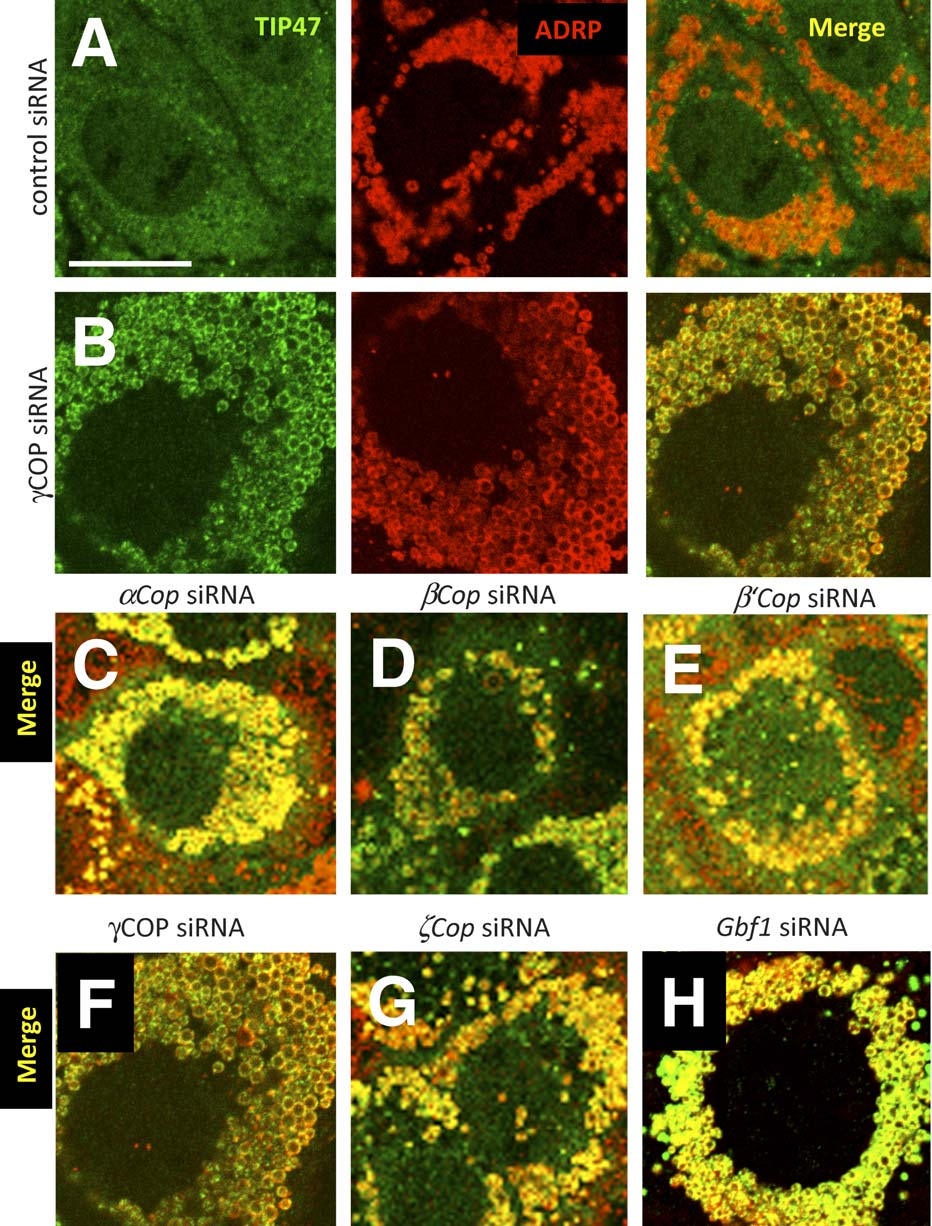
TIP47 Translocates to the Lipid Droplet Surface upon Down-Regulation of COPI Mouse AML12 cells were treated with ALLStars negative control siRNA (control) (A) or siRNAs targeting γCOP (B) for 4 d, respectively. Cells were incubated with 400 μM oleic acid for 12 h prior to staining with antibodies detecting TIP47 (green channel) or ADRP (red channel). Images of the separate channels as well as a merged image are shown. (C–H) Merged channel images of similarly treated mouse AML12 cells. Targeted genes are indicated. Scale bar in (A) represents 50 μm.

**Figure 12:**
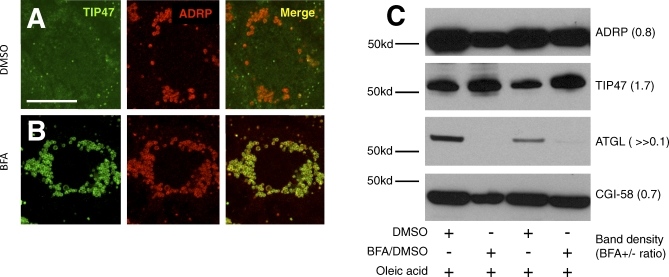
Changes in the Abundance of Selected Lipid Droplet Proteome Members Following BFA Treatment (A and B) Mouse AML12 cells were grown in the presence of NEFA (400 μM oleic acid) overnight and incubated in DMSO (A) or 5 μM BFA in DMSO (B) for 6 h, prior to staining with antibodies detecting TIP47 (green channel) or ADRP (red channel). Separate images for the different channels as well as a merged image are shown. (C) Western blots of lipid droplet protein preparations. Lipid droplets from AML12 cells grown in the presence of 400 μM oleic acid and treated with either DMSO (lanes one and three) or 5 μM BFA in DMSO (lanes two and four) were purified by sucrose gradient ultracentrifugation, and equal amounts of the associated proteins were subjected to western blot. Two separate experiment replicates are represented. Band density ratios (±BFA) are given. Scale bar in (A) represents 50 μm.

Wild-type COPI could mediate release of NEFA from lipid droplets by altering the heterogeneous and dynamic collection of lipid droplet-associated proteins found in different cell types and conditions [72]. To further explore what happens to lipid droplets following COPI knockdown, we examined the distribution of TIP47 and ADRP on the lipid droplet surface. These are the only PAT proteins expressed in AML12 cells [68]. In control cells incubated with oleic acid, and control siRNAs, ADRP was associated with the lipid droplet surface whereas TIP47 was mostly found in smaller punctate cytoplasmic inclusions and more ill-defined cytoplasmic locations ([68] and Figure 11A). TIP47 and ADRP were not colocalized in untreated cells. Following siRNA treatments targeting α, β, β′, γ, and ζ COPI subunit or *Gbf1* transcripts, both ADRP and TIP47 were observed on the same lipid droplets (Figure 11B–11H). Treating the cells with BFA had the same effect on TIP47 localization (Figure 12A and 12B). These data indicate that COPI is required for a wild-type pattern of PAT localization to the lipid droplet.

PAT proteins are tightly associated with the lipid droplet surface. In order to distinguish localization to the region of the lipid droplet from true localization to the lipid droplet surface, we treated cells with BFA after oleic acid feeding, and isolated lipid droplets by sucrose gradient ultracentrifugation. This treatment separates the lipid droplets from cytosol and other membrane fractions. To determine what proteins were on the lipid droplets, western blots were probed with antibodies detecting ADRP, TIP47, and ATGL, as well as the ATGL cofactor CGI-58. Whereas ADRP and CGI-58 remained quantitatively unchanged after BFA treatment, TIP47 protein levels in the lipid droplet fraction increased nearly 2-fold (Figure 12C). There was no change in TIP47 in the cytosolic fraction (unpublished). The cell-staining experiments showed a more dramatic increase in TIP47 at the ADRP-positive lipid droplets than we observed in the western blots, but importantly, both cell staining and western blotting show increased TIP47 on COPI inhibition. Strikingly, ATGL levels decreased to near or below the detection limit, suggesting that BFA treatment drives ATGL off the lipid droplet surface, or prevents ATGL association with the lipid droplet (Figure 12C). Thus, both cell staining and analysis of isolated droplets indicate that wild-type COPI limits abundance of TIP47 at the lipid droplet surface and is required for ATGL localization to the droplet surface. Taken together with the epistasis results demonstrating that COPI and ATGL function in the same pathway, these results indicate that COPI-mediated targeting of ATGL to the lipid droplet is required for lipolysis.

## Discussion

Positive regulation of lipolysis by the COPI retrograde-vesicle trafficking pathway was the most striking and unexpected result of our screen. We have found that interference with COPI function, either by RNAi or compounds, in *Drosophila* Kc_167_ or S3 cells, or in mouse 3T3-L1 or AML12 cells, results in increased lipid storage. Furthermore, recent and parallel studies in yeast [73] and *Drosophila* S2 cells [52] also suggested a role of COPI function in lipid droplet regulation. Interestingly, only the ɛ-subunit of the COPI complex failed to result in a lipid droplet deposition phenotype on knockdown. Although we cannot rule out limited RNAi efficacy or increased protein stability, ɛCOP was the only canonical COP subunit not resulting in a lipid storage phenotype in a parallel study using different cells and reagents [52], and we found that targeting of ɛCOP transcripts by RNAi in AML12 cells had a weak effect on lipid storage at best. Finally, ɛCOP is the only dispensable subunit in a recent study identifying COPI activity coupled with fatty acid biosynthesis as a host factor important for *Drosophila* C virus replication [74]. This is especially interesting, as certain enveloped viruses, including Hepatitis C virus, assemble on lipid droplets [75,76]. Taken together, these results indicate that six out of the seven wild-type COPI subunits mediate lipid storage by positively regulating lipolysis.

COPI could have a direct or indirect effect on lipid storage. The indirect mechanism is poorly defined, but if the Golgi is a “sink” for phospholipids derived from TG stores, then decreased Golgi function could simply decrease demand for TG substrate. If NEFA (from the media in fed cells, and from biosynthesis in unfed cells) conversion to TG continues, then increased lipid droplet volume would occur. It is also possible that canonical COPI function transporting lipids and proteins from the Golgi to the ER is ultimately responsible for lipid droplet utilization and protein composition at the lipid droplet surface. For example, COPI might be required for the particular phospholipid composition in hemimembranes formed on nascent droplets, which secondarily alter TIP47 and ATGL localization in mature lipid droplets.

However, evidence that Golgi function per se is not linked to lipid storage phenotypes, as well as direct association of COPI members and regulators with the lipid droplet or PAT proteins supports a more direct model. The COPI and COPII pathways have established roles as constitutive vesicle transport systems that cycle proteins as well as lipid from the Golgi to the ER (COPI), or vice versa (COPII) [37]. Interference with either of the COP trafficking systems results in disturbed ER and Golgi function [38,39]. The lipid overstorage phenotype was only seen in the case of interference with COPI trafficking. This indicates that the lipid overstorage phenotype is not a simple consequence of ER and Golgi function. Finally, in an indirect model in which COPI shuttles only between the Golgi and the ER, COPI should not be lipid droplet associated. However, COPI subunits are directly associated with the lipid droplet surface as shown by proteomics [6]. Additionally, Arf1 binds to ADRP, which is exclusively associated with the lipid droplet surface [77]. Arf79F, the *Drosophila* homolog of mammalian Arf1, also localizes to lipid droplets in *Drosophila* S2 cells [52].

**Figure 13:**
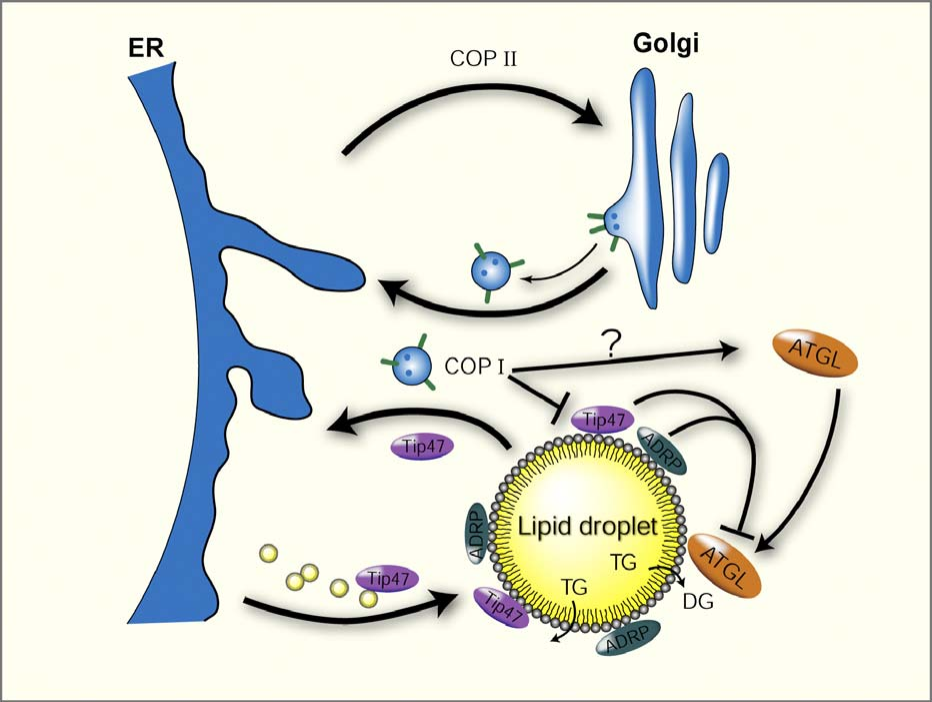
COPI Trafficking and Lipid Droplet Model COPI and COPII trafficking systems shuttle proteins and lipids between ER and Golgi. Small TIP47-coated lipid droplets are born at the ER and reside in the cytosol. The bigger lipid droplets are coated by ADRP, and TIP47 is returned to the ER. ATGL can translocate between the cytosol and the lipid droplet surface, where it begins the conversion of TG to DG and eventually NEFA. Other TG lipases may also be present (indicated by the second arrow liberating TG from the hydrophobic lipid droplet core). Wild-type Golgi–ER transport function of COPI might be necessary for proper lipid droplet biogenesis at the ER. COPI could directly function at the lipid droplet surface, mediating a similar ER-targeted transport function as reported for the Golgi–ER COPI transport. COPI is a positive regulator of ATGL with a unresolved mechanism (indicated by the question mark) and a negative regulator of TIP47 lipid droplet localization. ADRP and TIP47 are negative regulators of ATGL activity at the lipid droplet surface.

We propose that COPI is likely to function directly at the lipid droplet surface and not indirectly through the Golgi (Figure 13). Perhaps COPI is a destination-specific transporter returning lipid droplet surface hemimembrane and Golgi membrane to the ER. The transport system that brings nascent lipid droplets from the ER to the lipid droplet has not been elucidated, but it is intriguing that the transport/PAT protein TIP47 is found preferentially on small lipid droplets. Small lipid droplets derived from the ER are thought to help build larger droplets by fusion. TIP47-coated droplets might form in the ER, and then COPI could return TIP47 to the ER after the lipid cargo is deposited. In this model, TIP47 becomes trapped at the lipid droplet surface in the absence of COPI.

Although we observed increased TIP47 on ADRP-positive droplets by both western blot and cell staining, the cell staining result was more dramatic. Our model might also explain why. The punctate staining of TIP47 in untreated cells could be due to TIP47 on nascent droplets that might also cofractionate with the larger ADRP-positive droplets in the western blots, leading to a less dramatic enrichment for TIP47 relative to ADRP in that experiment. However, we cannot rule out other explanations, such as nonlinear detection of antigen concentration or epitope masking in the cell staining experiments.

COPI perturbation increases stored TG by decreasing the lipolysis rate (this study, [52]) indicating that the wild-type COPI complex promotes lipolysis. We have shown that COPI directly or indirectly removes TIP47 from the lipid droplet surface and promotes ATGL localization to the droplet surface, where lipolysis occurs. ATGL has a key role in lipid droplet utilization, and ATGL association with the droplet is reduced by ADRP and Tip47 [68]. Our epistasis experiments combining siRNA-mediated ATGL knockdown and BFA or Exo1 compound treatment demonstrated that the decrease in lipolysis rate is due to loss of ATGL activity. COPI activity specifically alters lipid droplet surface composition by increasing the amount of TIP47 and reducing the amount of ATGL at ADRP-coated lipid droplets. We suggest that COPI negatively regulates localization of TIP47. TIP47 in turn prevents ATGL localization. The rescue of the double-knockdown phenotype of TIP47 and ADRP by BFA or Exo1 suggests that COPI has an independent feed-forward effect on ATGL levels at the lipid droplet surface.

Although we have focused our attention here on COPI, our systematic and genome-wide exploration of gene functions required for lipid storage in *Drosophila* significantly increases experimental access to the complex molecular processes regulating lipid storage and utilization. Further, the use of multiple screens using different cell types and different organisms greatly increases confidence in the genes in the intersection. Given widespread concerns about RNAi screening efficacy and off-target effects, as well as the time and effort required for downstream analysis, systematic use of multiple species and libraries to address a single biological question might be cost effective in addition to resulting in more durable datasets. Primary screens in *Drosophila* cells followed by secondary screens in mouse cells are much less expensive than a similar genome-wide screen in mammalian cells. Additionally, the availability of mutants in most *Drosophila* genes, along with demonstrated translation to mammalian systems, provides a valuable entry point for in-depth analyses in both fly and mouse; and eventually for the selection of therapeutic targets for emerging problems associated with obesity and other metabolic disorders.

## Materials and Methods

### Genome-wide *Drosophila* RNAi screen wet-bench procedures.

We used the Harvard *Drosophila* RNAi Screening Center (DRSC, http://www.flyrnai.org) dsRNA collection, which covers more than 95% of the transcriptome (Release 3.2 BDGP) with a total of 17,076 dsRNAs [44] in duplicate. We seeded 1.5 × 10^4^ Kc_167_ cells (DRSC) in 10 μl of serum-free Schneider's medium (GIBCO) in each well of microscopy-quality 384-well plates containing the pre-aliquoted dsRNAs (approximately 250 ng of dsRNA/well). Plates were spun at 1,200 rpm for 1 min and incubated for 45 min at 25 °C. We then added 40 μl of complete Schneider's medium supplemented with 10% FCS (JRH Biosciences), 50 units penicillin; and 50 μg of streptomycin/ml (GIBCO) and ±400 μM oleic acid (Calbiochem) complexed to 0.4% BSA (Sigma). Plates were sealed and incubated in a humidified incubator at 25 °C for 4 d. The cells were subsequently fixed for 10 min in 4% formaldehyde in PBS followed by a 10-min permeabilization step in PBS including 0.1% Triton X-100. For lipid droplet visualization and cell counting (nuclei), we incubated for 1 h with PBS including 5 μg/ml BODIPY493/503 (Molecular Probes) and 5 μg/ml DAPI or 5 μg/ml Hoechst33342 (Molecular Probes). After two washes with PBS including 0.01% Tween-20, cells were kept in 40 μl of PBS and visualized with a 20× objective on a Discovery1 automated microscope system (Molecular Devices).

### Secondary RNAi screen wet-bench procedures.

A subset of 276 genes of the primary screen library were targeted by 362 additional dsRNAs (Table S10) generated from PCR products obtained from the *Drosophila* RNAi screening center of Harvard (DRSC). PCR fragments were reamplified using a modified T7 oligonucleotide (5′-GTA ATA CGA CTC ACT ATA GG-3′) and a touchdown PCR protocol. PCR products were subsequently used for in vitro transcription reactions using T7 RNA polymerase (Fermentas). Following DNAse-mediated digestion of the PCR template, dsRNAs were purified with Multiscreen PCR purification filter plates (Millipore). RNAi treatment was performed either as described for the primary screen in optical-quality 96-well plates (BD) with adjusted dsRNA and cell numbers, in duplicate (approximately 1 μg of dsRNA and 5 × 10^4^ cells/well). Imaging was performed either with a BD Pathway 855 Bioimager automated microscope (BD) or with a Zeiss Axiovert200M (Carl Zeiss) and the OpenLab software (Improvision).

For the secondary mouse siRNA screen (Table S10), we used AML12 murine liver cells (Steven Farmer, Boston University) and 3T3-L1 fibroblast cells (ATCC) grown according to protocols of the American Type Culture Collection (ATCC). Assays were done in 96-multiwell plates (Fisher Scientific) at a density of 0.25 × 10^4^ cells/well on growth medium supplemented with 200 μM oleic acid, which was added 18 h prior to fixation of the cells. Cells were transfected with Hiperfect transfection reagent (0.75 μl/well) (Qiagen) and experimental or ALLStars negative control siRNA oligonucleotides (10 nM), according to the manufacturer's instructions (Qiagen). Four days after transfection, cells were fixed and stained as described above for the *Drosophila* cells and imaged with a BD Pathway 855 Bioimager automated microscope system (BD).

### Image analysis.

Images of *Drosophila* cells (two sites/well in the primary screen; six sites/well for the secondary screen) were processed with a custom image segmentation algorithm (available from M. Beller upon request) written for the ImageJ software package [78]. After a sharpening and a brightness/contrast adjustment (for the BODIPY images; equal values for all images) or a gamma correction (for the DAPI images; same values for all images), a background subtraction followed by an Otsu thresholding step was run (Figure 1A–1D). Watershed processing to identify solitary particles followed. Finally, lipid droplets or nuclei were identified with the generic “analyze particles” function of the ImageJ software with the following settings: (1) settings for the nuclei: size from 10 to 10,000 pixels, 256 bins, outlines as well as measurement results displayed, measurements on the edges excluded, clear results, flood, and summary of the results; and (2) settings for the lipid droplets: identical parameters except size ranging from one to 200 pixels and a circularity from zero to one. For each detected particle, the size and area were measured. For each image, the total numbers of particles (“counts”) or cumulative measured area for all particles (“area”) are reported. A custom Perl script concatenated the summarized measurements, and the obtained information was used to calculate the ratio of lipid droplets per cells as a measure of lipid storage (“lipid droplet/nuclei (area)” or “lipid droplet/nuclei (counts)”).

Mammalian cell image analysis (four sites/well) was performed as described above with some adjusted settings reflecting the larger mammalian cell size as well as differences in imaging equipment (no brightness or contrast adjustments were applied). The generic “analyze particles” function of the ImageJ software was used with the following settings: (1) settings for the nuclei: size from 80 to 10,000 pixels, 256 bins, outlines as well as measurement results displayed, measurements on the edges excluded, clear results, flood, and summary of the results; and (2) settings for the lipid droplets: identical parameters, except the size ranging from one to 2,000 pixels and a circularity from zero to one.

### Primary screen data analysis.

The general thrust of the analysis is given below and is followed by a detailed description. Screen data are available (Table S4; http://lipofly.mpibpc.mpg.de/). Results were robust to data handling method (Table S1). Genes passing thresholding conditions (Tables S2 and S3) were used for the GO term analysis (Table S5). *B*-score *p*-values can be used to further restrict the gene lists shown in Tables S2 and S3.

Data analysis was performed with custom scripts written in the R language and packages provided by the Bioconductor project [79]. The lipid droplets and nuclei area measurements of the two images per well were used to calculate an averaged lipid area per nuclei area value per well. Additionally to the primary images, a number of wells required reimaging based on visual inspection (size of the complete dataset: *N* = 48,241 wells). To identify and extract images with bad quality, the values for lipid droplet (LD) area and count measurements as well as for the corresponding nuclei measurements of the two images per well were plotted against each other to look for variation within wells. In addition, the corresponding “LD area per nuclei area” and “LD count per nuclei count” ratios were plotted against each other per well. These plots showed 95 prominent outliers (segmentation artifacts/“bad” wells), which were removed (resulting *N* = 48,146 wells). The data values of reimaged wells were averaged.

The screen dataset was platewise normalized for within-plate and between-plate differences by four different algorithms. Because of the limited number of controls per plate, 98% of the wells per plate were used as a reference set in the normalization procedure as proposed in [47] in which the largest and smallest 1% values of the plate were removed to generate the reference set. Before data normalization, LD areas per nuclei area ratios were log-transformed. A classical robust *Z*-score normalization was performed first [zi = (xi − medianj)/madj, where zi is the *Z*-score of well *i*; xi is the raw value of well *i*; and medianj and madj are the median and median absolute deviation (MAD) of the plate j] in addition to the recently proposed strictly standardized mean difference normalization [SSMDi = (xi − meanj)/square root (2/nj − 2.5 × ((nj − 1) × SDj2))]. Those related algorithms were supplemented with both a fitted linear model normalization using the Prada package [45] and by *B*-score normalization [46]. Benjamini and Hochberg FDR-corrected *p*-values for all dsRNAs were calculated with the complete screen data (without the largest and smallest 1%) as a reference set. Scoring was done both on a platewise and screenwise manner. For the platewise hit identification, positives were identified by a quartile-based thresholding algorithm [48]. For this purpose, the first quartile (Q1), the median (Q2), and the third quartile (Q3) were calculated first. Afterwards, threshold *T* were calculated [*T*_upper_ = Q3 + *c* × (Q3 − Q2) and *T*_lower_ = Q1 − *c* × (Q2 − Q1), where *c* is a variable depending on the targeted error rate] [48]. The same hit selection strategy was also chosen for the screen-wide hit identification among the linear model normalized dataset. For the other normalization algorithms, fixed thresholds were selected. In all cases, threshold levels (as well as the *c* in the quartile-based thresholding) were chosen based on the identification rates of the internal controls *brummer* dsRNA, *midway* dsRNA, and wells with no oleic acid, which were present on every screening plate. The highest possible threshold was chosen capable of balancing both false-positive and -negative rates.

Identified *Drosophila* lipid regulating gene functions (Tables S2 and S3) were subjected to in silico analysis for enriched GO terms. For this purpose, we used the standard settings of the VLAD tool (Mouse Genome Informatics Web site [51]) using the complete *Drosophila* genome as a reference set. Results of the enrichment analyses were visualized by pruned GO term networks (pruning threshold = 4; collapsing threshold = 5), and results (pruning threshold = 3; collapsing threshold = 6) are additionally tabulated (Table S5).

Detailed lists of the scoring genes were annotated with the following information (Table S9): GO terms from FlyBase [80]; orthologs from FlyMine [81]; human disease gene orthologs from Homophila (http://superfly.ucsd.edu/homophila/, used with a significance threshold of *E* < 1 × 10–50, [31]; InParanoid [82] orthologs (http://inparanoid.sbc.su.se/cgi-bin/index.cgi); and *Drosophila* [4,33]; as well as mammalian [5,6,32,34] lipid droplet subproteome data.

### Secondary screen data analysis.

A subset of genes identified in the genome-wide screen with a potential function in cellular lipid storage regulation was assayed by at least one additional dsRNA. In total, 276 genes were tested by targeting with 362 dsRNA sequences (Table S10). Because we were interested in validating the full range of phenotypes observed and not just the positives, we sampled across a broad range of *B*-scores. We performed directed retesting on the genes encoding COPI members. To test for COPI specificity, we used secondary dsRNA sequences targeting Arf family members not involved in COPI function as well as COPII vesicle transport encoding transcripts as controls. dsRNAs targeting those genes did not result in a phenotype in the primary screen. For a “positive” identification, we required that two independent nonoverlapping dsRNAs or siRNAs give the same phenotype. In addition, we tested mouse AML12 hepatocytes and mouse 3T3-L1 fibroblasts for an evolutionary conservation of the identified lipid storage modulators. Assuming that off-target effects are random, this also minimizes misleading off-target effects, and is certainly more stringent than the current standard of two positive RNAi reagents with retesting in the same species and cell type [60]. In total, 127 mouse genes covered by 312 siRNAs were tested (Table S10). Genes across the screen that were validated using the image-based analysis with additional RNAi reagents are listed in Table S7. Additional gene and COPI validation comes from small compound phenocopy, cell staining experiments, and measurements on lipid metabolism as outlined further below.

Lipid droplet area and nuclei area measurements obtained from the image segmentation procedure, which was carried out as described for the primary screen results, was used to express the ratio of lipid per cell. For each screen, plate data were median normalized. In order to identify genes modulating lipid storage, a basic thresholding of median ± 2 × MAD was used. Since the datasets were enriched for modulators of lipid storage, the median as well as MAD was calculated on the basis of control wells incorporated in the assay plates. For the *Drosophila*, AML12, and 3T3-L1 datasets, those wells contained no RNAi reagent, but were otherwise treated identical to the experimental wells. Screening plates also contained other control dsRNAs/siRNAs wells. The *Drosophila* secondary screen plates contained wells with dsRNAs targeting *bmm* or *mdy* as in the primary screen. In the case of the 3T3-L1 and AML12 cells, plates contained siRNAs targeting *Atgl* or a combination of two siRNAs targeting both *Adrp* and *Tip47* transcripts [68]. Median ± thresholds were adjusted in order to fulfill the same prerequisites as in the primary screen, namely a maximum of identified controls with a minimum of false positives. False positives were scored based on the wells lacking RNAi reagent.

### Small-molecule compound-based modulation of cellular lipid storage.

Small-molecule compound experiments were performed with embryonic *Drosophila* S3 cells (Bloomington *Drosophila* Stock Center [DGRC]), which showed excellent oleic acid feeding characteristics during RNAi assay development but inferior RNAi characteristics as compared to the Kc_167_ cells. S3 cells showed superior adherence during automated liquid handling in 1,536-well format. We dispensed 4 μl of cells at 1.25 × 10^6^ cells/ml into LoBase Aurora COC 1,536-well plates (black walled, clear bottom) with a bottle-valve solenoid-based dispenser (Aurora) to obtain 5,000 cells/well. A total of 23 nl of compound solution of different concentrations were transferred to the assay plates using a Kalypsis pin tool equipped with a 1,536-pin array containing 10-nl slotted pins (FP1S10, 0.457-mm diameter, 50.8 mm long; V&P Scientific). One microliter of oleic acid (400 μM) was added, and the plate was lidded with stainless steel rubber gasket-lined lids containing pinholes. After 18–24-h incubation at 24 °C and 95% humidity, BODIPY 493/503 (Molecular Probes) was added to the wells to stain lipid droplets, and the Cell Tracker Red CMTPC dye (Molecular Probes) was added to enumerate cell number. Fluorescence was detected by excitation of the fluorophores with a 488-nm laser on an Acumen Explorer (TTP Lab Tech). The total intensity in channel 1 (500–530 nm) reflected lipid droplet accumulation. Cells were detected using channel 3 (575–640 nm) with 5-μm width and 100-μm depth filters. The ratio of the total intensity in PMT channel 1 over total intensity of channel 3 was also calculated. Percent activity was computed relative to an internal control (100% inhibited lipid droplet deposition due to the presence of 20 μM Triacsin C), which was added to 32 wells/plate.

### Lipolysis and lipogenesis measurements in AML12 cells.

Measurements of NEFA released from lipid droplets or incorporated into the TG fraction were performed as previously described [23,68,83]. Briefly, AML12 cells treated with or without specific siRNAs (10 nM) for 4 d were incubated overnight with growth medium supplemented with 400 μM oleic acid complexed to 0.4% bovine serum albumin to promote triacylglycerol deposition and [3H] oleic acid, at 1 × 10^6^ dpm/well, was included as a tracer. In lipolysis experiments, re-esterification of fatty acids in AML12 cells was prevented by including 10 μM Triacsin C (Biomol), an inhibitor of acyl coenzyme A synthetase [67], in the medium. Quadruplicate wells were tested for each condition. Lipolysis was determined by measuring radioactivity released into the media in 1 h. For the lipid extraction and thin layer chromatography, the cell monolayer was washed with ice-cold PBS and scraped into 1 ml of PBS. Lipids were extracted by the Bligh-Dyer method [84], and 10% of the total lipid was analyzed by thin layer chromatography [83,85]. AML12 cells treated with or without specific siRNAs were additionally incubated with either vehicle (DMSO), 5 μM of Exo1 (12.5 mg/ml DMSO), or BFA (10 mg/ml DMSO) during the time of radioactivity release into the media (2 h). NEFA incorporation into the TG fraction and NEFA release are calculated as nanomoles/milligram protein (Table S8). Protein measurements were performed using a commercial BCA assay kit (Pierce Biotechnology) according to the manufacturer's instructions. Statistical significance was tested by impaired Student *t* test (GraphPad software).

### Antibodies.

Rabbit anti-TIP47 and goat anti-ADRP were used as previously published [9]. Antibodies targeting mouse ATGL were purchased from Cell Signaling Technology. The CGI-58 antibody was a gift from Dr. Osumi [29].

### Immunocytochemistry.

Cells were plated in four-well Lab-Tek chamber slides (Nunc) and incubated overnight with 400 μM oleic acid. In compound experiments, wells received vehicle (DMSO) or 5 μM BFA (10 mg/ml DMSO) treatment for 6 h. RNAi treatment prior to immunocytochemistry is outlined above. For ADRP and TIP47 staining, cells were fixed in 3% v/v paraformaldhyde/PBS for 15 min at room temperature. Staining was performed by published methods [9,86]. Cells were viewed with a confocal laser scanning microscope (LSM510; Carl Zeiss MicroImaging) using a 63× oil objective lens.

### Fat cake preparation.

Eight 100-mm dishes for each condition were treated with 400 μM oleic acid overnight and further treated with DMSO or BFA (5 μM) for 6 h on the next day. Cells were washed three times with phosphate buffered saline (PBS; pH 7.4), scraped into PBS, and then pelleted by low-speed centrifugation. LD isolation was as reported [8]. The lipid fat cake was isolated and resuspended in 150 μl of PBS containing 5% SDS before 150 μl of 2× Laemmli sample buffer were added. For CGI-58 and ATGL western blots, those protein extracts were directly loaded. For ADRP and Tip47, the samples were diluted 200-fold (ADRP) or 20-fold (TIP47), respectively. A total of 35 μl were loaded then on each lane. X-ray films were used to detect the western blots. Quantification was done with ImageJ [78].

## Supporting Information

Table S1 Comparison of Different Primary Screen Analysis Methods (34 KB XLS)

Table S2 Genes Showing an Understorage Phenotype in the Primary *Drosophila* RNAi Screen (187 KB DOC)

Table S3 Genes Showing an Overstorage Phenotype in the Primary *Drosophila* RNAi Screen (273 KB DOC)

Table S4 *Drosophila* Primary Screen Dataset (16 MB XLS)

Table S5 Geneontology-Term Analysis with the VLAD Tool (186 KB XLS)

Table S6 Detailed Comparison to the Study of Guo et al. [52] (72 KB XLS)

Table S7 Validated RNAi Screen Hits (47 KB XLS)

Table S8 AML12 Cell Lipolysis and Lipogenesis Measurements (31 KB XLS)

Table S9 *Drosophila* Primary Screen Hits (1 MB XLS)

Table S10 Secondary screen data (848 KB XLS)

## Accession numbers

*Drosophila* RNAi screen hits: FBgn0000028, FBgn0000042, FBgn0000114, FBgn0000339, FBgn0000489, FBgn0000547, FBgn0000567, FBgn0001186, FBgn0001204, FBgn0001301, FBgn0002878, FBgn0003048, FBgn0003118, FBgn0003339, FBgn0003380, FBgn0003392, FBgn0003462, FBgn0003557, FBgn0003607, FBgn0003691, FBgn0004167, FBgn0004187, FBgn0004401, FBgn0004587, FBgn0004595, FBgn0004611, FBgn0004652, FBgn0004797, FBgn0004838, FBgn0004856, FBgn0004879, FBgn0005411, FBgn0005626, FBgn0005630, FBgn0010083, FBgn0010215, FBgn0010355, FBgn0010638, FBgn0010750, FBgn0011571, FBgn0011701, FBgn0013746, FBgn0014020, FBgn0015320, FBgn0015818, FBgn0015919, FBgn0016926, FBgn0016940, FBgn0019643, FBgn0020611, FBgn0020908, FBgn0021768, FBgn0022246, FBgn0023143, FBgn0024285, FBgn0024308, FBgn0024555, FBgn0024754, FBgn0025638, FBgn0026206, FBgn0026317, FBgn0026620, FBgn0026722, FBgn0026878, FBgn0027495, FBgn0027589, FBgn0027885, FBgn0027951, FBgn0028360, FBgn0028420, FBgn0028982, FBgn0029123, FBgn0029526, FBgn0029661, FBgn0029731, FBgn0029766, FBgn0029824, FBgn0029850, FBgn0029873, FBgn0029935, FBgn0030075, FBgn0030077, FBgn0030087, FBgn0030093, FBgn0030189, FBgn0030244, FBgn0030390, FBgn0030434, FBgn0030492, FBgn0030608, FBgn0030872, FBgn0030904, FBgn0031008, FBgn0031030, FBgn0031031, FBgn0031074, FBgn0031093, FBgn0031232, FBgn0031390, FBgn0031518, FBgn0031626, FBgn0031673, FBgn0031816, FBgn0031836, FBgn0031888, FBgn0031894, FBgn0032049, FBgn0032340, FBgn0032351, FBgn0032360, FBgn0032363, FBgn0032388, FBgn0032454, FBgn0032622, FBgn0032800, FBgn0032868, FBgn0032945, FBgn0033155, FBgn0033160, FBgn0033541, FBgn0034071, FBgn0034402, FBgn0034646, FBgn0034709, FBgn0034839, FBgn0034946, FBgn0034967, FBgn0035085, FBgn0035136, FBgn0035294, FBgn0035546, FBgn0035569, FBgn0035631, FBgn0036274, FBgn0036374, FBgn0036470, FBgn0036556, FBgn0036734, FBgn0036761, FBgn0036811, FBgn0037024, FBgn0037149, FBgn0037178, FBgn0037250, FBgn0037278, FBgn0037304, FBgn0037568, FBgn0037920, FBgn0037924, FBgn0038168, FBgn0038191, FBgn0038343, FBgn0038359, FBgn0038391, FBgn0038592, FBgn0038633, FBgn0038662, FBgn0039054, FBgn0039941, FBgn0039959, FBgn0039997, FBgn0040279, FBgn0040291, FBgn0040369, FBgn0040534, FBgn0040651, FBgn0040777, FBgn0042693, FBgn0050126, FBgn0050470, FBgn0051313, FBgn0051374, FBgn0051632, FBgn0051814, FBgn0052056, FBgn0052062, FBgn0052112, FBgn0052121, FBgn0052150, FBgn0052202, FBgn0052352, FBgn0052397, FBgn0052440, FBgn0052635, FBgn0052704, FBgn0052710, FBgn0052711, FBgn0052970, FBgn0053207, FBgn0053500, FBgn0053516, FBgn0058413, FBgn0061200, FBgn0083976, FBgn0083992, FBgn0085381, FBgn0086441, FBgn0086674, FBgn0086899, FBgn0243486, FBgn0259162, FBgn0259169, FBgn0259171, FBgn0259217, FBgn0259228, FBgn0259240, FBgn0259243, FBgn0000008, FBgn0000100, FBgn0000116, FBgn0000212, FBgn0000409, FBgn0000492, FBgn0000636, FBgn0000986, FBgn0001133, FBgn0001216, FBgn0001217, FBgn0001218, FBgn0001942, FBgn0002023, FBgn0002590, FBgn0002593, FBgn0002607, FBgn0002906, FBgn0002921, FBgn0003031, FBgn0003060, FBgn0003209, FBgn0003277, FBgn0003279, FBgn0003360, FBgn0003600, FBgn0003687, FBgn0003701, FBgn0003941, FBgn0003942, FBgn0004110, FBgn0004922, FBgn0004926, FBgn0005593, FBgn0005614, FBgn0005630, FBgn0005648, FBgn0008635, FBgn0010078, FBgn0010220, FBgn0010348, FBgn0010352, FBgn0010391, FBgn0010409, FBgn0010410, FBgn0010412, FBgn0010431, FBgn0010612, FBgn0010808, FBgn0011211, FBgn0011272, FBgn0011284, FBgn0011701, FBgn0011726, FBgn0011745, FBgn0011837, FBgn0012034, FBgn0013275, FBgn0013276, FBgn0013277, FBgn0013278, FBgn0013279, FBgn0013325, FBgn0013981, FBgn0014020, FBgn0014857, FBgn0015024, FBgn0015288, FBgn0015393, FBgn0015756, FBgn0015774, FBgn0015778, FBgn0015834, FBgn0016120, FBgn0016694, FBgn0016926, FBgn0017397, FBgn0017545, FBgn0017566, FBgn0017579, FBgn0019624, FBgn0019886, FBgn0019936, FBgn0020129, FBgn0020386, FBgn0020439, FBgn0020910, FBgn0022343, FBgn0022935, FBgn0023170, FBgn0023171, FBgn0023213, FBgn0023531, FBgn0024150, FBgn0024330, FBgn0024733, FBgn0024939, FBgn0025286, FBgn0025582, FBgn0025724, FBgn0025725, FBgn0026262, FBgn0026666, FBgn0026741, FBgn0027321, FBgn0027348, FBgn0027615, FBgn0028530, FBgn0028867, FBgn0028968, FBgn0028969, FBgn0029088, FBgn0029161, FBgn0029504, FBgn0029761, FBgn0029799, FBgn0029822, FBgn0029860, FBgn0029897, FBgn0030025, FBgn0030088, FBgn0030174, FBgn0030259, FBgn0030341, FBgn0030384, FBgn0030386, FBgn0030606, FBgn0030610, FBgn0030669, FBgn0030692, FBgn0030696, FBgn0030726, FBgn0030915, FBgn0030951, FBgn0030990, FBgn0031300, FBgn0031392, FBgn0031545, FBgn0031696, FBgn0031771, FBgn0031842, FBgn0031980, FBgn0032053, FBgn0032215, FBgn0032261, FBgn0032330, FBgn0032400, FBgn0032518, FBgn0032587, FBgn0032596, FBgn0032619, FBgn0032656, FBgn0032675, FBgn0032833, FBgn0032987, FBgn0033029, FBgn0033081, FBgn0033085, FBgn0033282, FBgn0033313, FBgn0033341, FBgn0033368, FBgn0033379, FBgn0033403, FBgn0033591, FBgn0033652, FBgn0033699, FBgn0033902, FBgn0033912, FBgn0034020, FBgn0034258, FBgn0034487, FBgn0034488, FBgn0034537, FBgn0034579, FBgn0034649, FBgn0034751, FBgn0034902, FBgn0034948, FBgn0034968, FBgn0034987, FBgn0035276, FBgn0035315, FBgn0035422, FBgn0035562, FBgn0035563, FBgn0035638, FBgn0035699, FBgn0035753, FBgn0035872, FBgn0035976, FBgn0036135, FBgn0036213, FBgn0036288, FBgn0036343, FBgn0036351, FBgn0036360, FBgn0036398, FBgn0036449, FBgn0036462, FBgn0036492, FBgn0036532, FBgn0036534, FBgn0036576, FBgn0036613, FBgn0036728, FBgn0036820, FBgn0036825, FBgn0036895, FBgn0036990, FBgn0037010, FBgn0037028, FBgn0037093, FBgn0037097, FBgn0037098, FBgn0037102, FBgn0037207, FBgn0037249, FBgn0037270, FBgn0037356, FBgn0037415, FBgn0037429, FBgn0037529, FBgn0037546, FBgn0037559, FBgn0037566, FBgn0037610, FBgn0037637, FBgn0037752, FBgn0037813, FBgn0037912, FBgn0037942, FBgn0037955, FBgn0038049, FBgn0038074, FBgn0038131, FBgn0038281, FBgn0038345, FBgn0038538, FBgn0038628, FBgn0038629, FBgn0038734, FBgn0038760, FBgn0038881, FBgn0038996, FBgn0039205, FBgn0039214, FBgn0039302, FBgn0039359, FBgn0039402, FBgn0039404, FBgn0039464, FBgn0039520, FBgn0039580, FBgn0039857, FBgn0040007, FBgn0040010, FBgn0040233, FBgn0040512, FBgn0040529, FBgn0040634, FBgn0040766, FBgn0040793, FBgn0043001, FBgn0043904, FBgn0050007, FBgn0050290, FBgn0050387, FBgn0051158, FBgn0051284, FBgn0051291, FBgn0051302, FBgn0051354, FBgn0051361, FBgn0051450, FBgn0051453, FBgn0051554, FBgn0051613, FBgn0051754, FBgn0051774, FBgn0051847, FBgn0052050, FBgn0052105, FBgn0052179, FBgn0052193, FBgn0052219, FBgn0052311, FBgn0052600, FBgn0052633, FBgn0052720, FBgn0052733, FBgn0052773, FBgn0052778, FBgn0052797, FBgn0053128, FBgn0053147, FBgn0053256, FBgn0053271, FBgn0053300, FBgn0053319, FBgn0058337, FBgn0062412, FBgn0062413, FBgn0083950, FBgn0085392, FBgn0085408, FBgn0085424, FBgn0085436, FBgn0086710, FBgn0086712, FBgn0086758, FBgn0086904, FBgn0250791, FBgn0250814, FBgn0250834, FBgn0250908, FBgn0259113, FBgn0259212, FBgn0259232, and FBgn0259246.

Mouse genes with a confirmed function in lipid storage regulation: *MGI:107807*, *MGI:107851*, *MGI:1333825*, *MGI:1334462*, *MGI:1335073*, *MGI:1351329*, *MGI:1353495*, *MGI:1354962*, *MGI:1858696*, *MGI:1861607*, *MGI:1891824*, *MGI:1891829*, *MGI:1913585*, *MGI:1914062*, *MGI:1914103*, *MGI:1914144*, *MGI:1914234*, *MGI:1914454*, *MGI:1915822*, *MGI:1916296*, *MGI:1917599*, *MGI:1929063*, *MGI:2385261*, *MGI:2385656*, *MGI:2387591*, *MGI:2388481*, *MGI:2443241*, *MGI:3041174*, *MGI:3694697*, *MGI:88192*, *MGI:95301*, *MGI:98342*, and *MGI:99431*.

## Abbreviations

ADRP: adipocyte differentiation related protein
ATGL: adipocyte triglyceride lipase
BFA: Brefeldin A
CGI-58: Comparative Gene Identification-58
COPI: Coat Protein Complex I
DG: diglyceride
*DRSC*: *Drosophila* RNAi Screening Center
dsRNA: double-stranded RNA
ER: endoplasmic reticulum
FDR: false discovery rate
GEF: guanine nucleotide exchange factor
GO: Gene Ontology
HSL: hormone sensitive lipase
LSD: lipid storage droplet
NEFA: non-esterified free fatty acid
PAT: perilipin, adipocyte differentiation related protein, tail interacting protein of 47 kDa
RNAi: RNA interference
TG: triglyceride
TIP47: tail interacting protein of 47 kDa
