# Supplementary material for: COPI Complex Is a Regulator of Lipid Homeostasis
Source: PLoS Biol. 2008 Nov 25;6(11):e292. doi: 10.1371/journal.pbio.0060292 (PMC2586367; doi:10.1371/journal.pbio.0060292)
Supplement: Table S2 — (187 KB DOC) [file pbio.0060292.st002.doc]

**Table S2: Genes showing an understorage phenotype in the primary *Drosophila* RNAi screen.**

Given are the *Drosophila* gene symbol, the average B-score normalized LD area per nuclei area value of the wells reaching the thresholding criteria (average B.Score) and the average Benjamini and Hochberg corrected p-value of the corresponding Amplicon(s) targeting the gene (note: a p-value greater than 0.05 might indicate a) additional, non-scoring wells or b) additional, non-scoring Amplicons targeting the given gene; B.Score.pVal). Additionally, the Flybase identifier (Flybase_id) is included.

| ***Drosophila* Gene Symbol** | **average B.Score** | **B.Score.pVal** | **Flybase_id** |
| --- | --- | --- | --- |
| *pxb* | -6.19077 | 4.95E-06 | FBgn0053207 |
| *CG32629* | -5.86579 | 2.32E-09 | FBgn0052629 |
| *CG11985* | -5.72962 | 6.22E-09 | FBgn0040534 |
| *flfl* | -5.69131 | 7.96E-09 | FBgn0024555 |
| *CG14710* | -5.60024 | 1.48E-08 | FBgn0037920 |
| *CG14712* | -5.47243 | 3.53E-08 | FBgn0037924 |
| *CG32970* | -5.4296 | 4.60E-08 | FBgn0052970 |
| *CG31814* | -5.38248 | 6.46E-08 | FBgn0051814 |
| *CG9925* | -5.33635 | 8.87E-08 | FBgn0038191 |
| *omd* | -5.25317 | 1.59E-07 | FBgn0038168 |
| *smi21F* | -4.93364 | 1.26E-06 | FBgn0016926 |
| *CG32150* | -4.92801 | 1.30E-06 | FBgn0052150 |
| *Csk* | -4.89345 | 1.63E-06 | FBgn0037925 |
| *Rho1* | -4.75644 | 0.33718 | FBgn0014020 |
| *CG17689* | -4.67819 | 5.90E-06 | FBgn0036374 |
| *Roc1a* | -4.57402 | 1.09E-05 | FBgn0025638 |
| *CG31374* | -4.44119 | 2.30E-05 | FBgn0051374 |
| *CG15084* | -4.28037 | 0.023256 | FBgn0034402 |
| *CG1845* | -4.26069 | 6.16E-05 | FBgn0033155 |
| *dpr3* | -4.07116 | 0.00016 | FBgn0053516 |
| *pros* | -4.05237 | 0.000176 | FBgn0004595 |
| *MTA1-like* | -4.04962 | 0.000177 | FBgn0027951 |
| *Scr* | -3.96019 | 0.000279 | FBgn0003339 |
| *CG32710* | -3.92271 | 0.000336 | FBgn0052710 |
| *CG32013* | -3.89908 | 0.000373 | FBgn0052013 |
| *CG30470* | -3.84358 | 0.001189 | FBgn0050470 |
| *snRNP69D* | -3.83237 | 0.000506 | FBgn0016940 |
| *CG15470* | -3.7335 | 0.000807 | FBgn0029731 |
| *CG32062* | -3.72204 | 0.012152 | FBgn0052062 |
| *upd2* | -3.70304 | 0.121468 | FBgn0030904 |
| *Sh* | -3.70217 | 0.000936 | FBgn0003380 |
| *mdy* | -3.67907 | 0.001044 | FBgn0004797 |
| *th* | -3.66575 | 0.018802 | FBgn0003691 |
| *l(1)G0148* | -3.57212 | 0.346007 | FBgn0028360 |
| *tlk* | -3.55749 | 0.001831 | FBgn0026698 |
| *CG14871* | -3.50052 | 0.00236 | FBgn0038343 |
| *Vha44* | -3.4887 | 0.002478 | FBgn0020611 |
| *UbcD2* | -3.46456 | 0.002732 | FBgn0015320 |
| *hkb* | -3.4011 | 0.493566 | FBgn0001204 |
| *kel* | -3.40076 | 0.003628 | FBgn0001301 |
| *CG4365* | -3.39785 | 0.442749 | FBgn0037024 |
| *CG31632* | -3.38424 | 0.003851 | FBgn0051632 |
| *Spt6* | -3.33777 | 0.000711 | FBgn0028982 |
| *CG17167* | -3.3326 | 0.004765 | FBgn0039941 |
| *CG11050* | -3.29472 | 0.005607 | FBgn0031836 |
| *MED19* | -3.28038 | 0.006402 | FBgn0036761 |
| *CG1113* | -3.25232 | 0.006677 | FBgn0037304 |
| *CG13830* | -3.23637 | 0.00713 | FBgn0039054 |
| *CG18599* | -3.23259 | 0.007226 | FBgn0038592 |
| *Pka-C3* | -3.23225 | 0.007226 | FBgn0000489 |
| *CG31650* | -3.19356 | 0.527878 | FBgn0031673 |
| *aret* | -3.17999 | 0.034675 | FBgn0000114 |
| *CG32711* | -3.17782 | 0.009025 | FBgn0052711 |
| *CG7042* | -3.13187 | 0.010758 | FBgn0035105 |
| *CG32440* | -3.13044 | 0.010791 | FBgn0052440 |
| *CG33305* | -3.12577 | 0.010893 | FBgn0032351 |
| *Smr* | -3.11235 | 0.002033 | FBgn0024308 |
| *pnt* | -3.06719 | 0.013529 | FBgn0003118 |
| *Sod* | -3.04704 | 0.014681 | FBgn0003462 |
| *CG40413* | -3.03203 | 0.015398 | FBgn0058413 |
| *Eip74EF* | -3.01971 | 0.022444 | FBgn0000567 |
| *CG8010* | -3.00198 | 0.017354 | FBgn0031008 |
| *Su(dx)* | -2.98724 | 0.018053 | FBgn0003557 |
| *Kr-h1* | -2.9849 | 0.018074 | FBgn0028420 |
| *CG17665* | -2.97727 | 0.018593 | FBgn0039997 |
| *Hex-A* | -2.96026 | 0.00127 | FBgn0001186 |
| *CG5830* | -2.94406 | 0.020999 | FBgn0036556 |
| *dalao* | -2.91604 | 0.99905 | FBgn0030093 |
| *Pvf2* | -2.91421 | 0.023256 | FBgn0031888 |
| *CG32352* | -2.89333 | 0.024873 | FBgn0052352 |
| *CG13095* | -2.88818 | 0.025301 | FBgn0032049 |
| *Flo* | -2.88272 | 0.025831 | FBgn0024754 |
| *CG14290* | -2.87997 | 0.026018 | FBgn0038662 |
| *CG13540* | -2.8649 | 0.027474 | FBgn0034839 |
| *snRNA:U1:95Cc* | -2.84894 | 0.029046 | FBgn0004187 |
| *lola* | -2.8451 | 0.029453 | FBgn0005630 |
| *CG15365* | -2.82845 | 0.031226 | FBgn0030077 |
| *Tao-1* | -2.8232 | 0.767166 | FBgn0031030 |
| *Srp54* | -2.82257 | 0.031784 | FBgn0024285 |
| *CG5614* | -2.81898 | 0.223132 | FBgn0038359 |
| *CG10473* | -2.81821 | 0.03222 | FBgn0032745 |
| *CG4400* | -2.79061 | 0.028107 | FBgn0030434 |
| *SmB* | -2.79023 | 0.034846 | FBgn0010083 |
| *CG2157* | -2.76586 | 0.094865 | FBgn0030244 |
| *Spx* | -2.76179 | 0.03844 | FBgn0015818 |
| *alien* | -2.7529 | 0.039693 | FBgn0013746 |
| *Lsd-2* | -2.72626 | 0.032999 | FBgn0030608 |
| *CG1074* | -2.69105 | 0.275824 | FBgn0037250 |
| *caz* | -2.67076 | 0.050855 | FBgn0011571 |
| *CG1017* | -2.66991 | 0.050855 | FBgn0035294 |
| *drosha* | -2.6665 | 0.051345 | FBgn0026722 |
| *repo* | -2.66262 | 0.05194 | FBgn0011701 |
| *CG8378* | -2.66167 | 0.05194 | FBgn0027495 |
| *CG7502* | -2.65157 | 0.05355 | FBgn0030987 |
| *CG32814* | -2.64054 | 0.055201 | FBgn0052814 |
| *CG6905* | -2.63647 | 0.05578 | FBgn0035136, FBgn0040291 |
| *CG8665* | -2.63195 | 0.056682 | FBgn0032945 |
| *CG12720* | -2.61169 | 0.059774 | FBgn0030379 |
| *CG17514* | -2.60963 | 0.060069 | FBgn0039959 |
| *CG3918* | -2.60682 | 0.635809 | FBgn0029873 |
| *CG15876* | -2.59538 | 0.062036 | FBgn0035569 |
| *pcx* | -2.58705 | 0.06363 | FBgn0003048 |
| *CG15631* | -2.58601 | 0.063783 | FBgn0031626 |
| *CG8405* | -2.58194 | 0.064683 | FBgn0034071 |
| *CG7564* | -2.57999 | 0.064968 | FBgn0036734 |
| *CG4496* | -2.55529 | 0.070961 | FBgn0031894 |
| *atms* | -2.5381 | 0.228439 | FBgn0010750 |
| *CG11107* | -2.53509 | 0.075631 | FBgn0033160 |
| *CG11345* | -2.53327 | 0.076025 | FBgn0035546 |
| *CG14926* | -2.52032 | 0.079092 | FBgn0032360 |
| *Ucp4A* | -2.50592 | 0.207438 | FBgn0030872 |
| *CG15364* | -2.50563 | 0.081652 | FBgn0030075 |
| *PP2A-B'* | -2.49726 | 0.083998 | FBgn0042693 |
| *CG6686* | -2.49278 | 0.085289 | FBgn0032388 |
| *CG32635* | -2.4757 | 0.326936 | FBgn0052635 |
| *Pep* | -2.47052 | 0.05372 | FBgn0004401 |
| *Hrb27C* | -2.46918 | 0.091688 | FBgn0004838 |
| *CG3074* | -2.4643 | 0.093073 | FBgn0034709 |
| *CG4325* | -2.45762 | 0.094865 | FBgn0026878 |
| *mei-P26* | -2.4544 | 0.136839 | FBgn0026206 |
| *Bx42* | -2.44808 | 0.078269 | FBgn0004856 |
| *Aac11* | -2.44107 | 0.099556 | FBgn0027885 |
| *Rae1* | -2.43936 | 0.10001 | FBgn0034646 |
| *B52* | -2.4216 | 0.657112 | FBgn0004587 |
| *CG15784* | -2.41856 | 0.106328 | FBgn0029766 |
| *ed* | -2.41584 | 0.106897 | FBgn0000547 |
| *CG14767* | -2.39777 | 0.111948 | FBgn0040777 |
| *CG15455* | -2.38949 | 0.114238 | FBgn0031121 |
| *GATAe* | -2.3879 | 0.405682 | FBgn0038391 |
| *caup* | -2.36069 | 0.124303 | FBgn0015919 |
| *fru* | -2.35776 | 0.468353 | FBgn0004652 |
| *PFE* | -2.35241 | 0.12713 | FBgn0032661 |
| *Plc21C* | -2.35072 | 0.127338 | FBgn0004611 |
| *CG33500* | -2.31547 | 0.139543 | FBgn0053500 |
| *C3G* | -2.3088 | 0.140693 | FBgn0026145 |
| *CG6509* | -2.28595 | 0.149601 | FBgn0032363 |
| *CG32397* | -2.27789 | 0.152441 | FBgn0052397 |
| *CG30126* | -2.27419 | 0.15401 | FBgn0050126 |
| *CG4328* | -2.2723 | 0.154826 | FBgn0036274 |
| *CG13377* | -2.26615 | 0.156916 | FBgn0040369 |
| *cni* | -2.26409 | 0.157212 | FBgn0000339 |
| *CG14303* | -2.25593 | 0.159446 | FBgn0038633 |
| *CG18166* | -2.25587 | 0.159446 | FBgn0029526 |
| *CG15754* | -2.25319 | 0.210791 | FBgn0030492 |
| *CG3065* | -2.24601 | 0.163078 | FBgn0034946 |
| *Act5C* | -2.23949 | 0.165943 | FBgn0000042 |
| *CG14446* | -2.23252 | 0.168263 | FBgn0029850 |
| *CG14561* | -2.2325 | 0.168263 | FBgn0037149 |
| *plx* | -2.22304 | 0.325192 | FBgn0004879 |
| *skpE* | -2.218 | 0.173774 | FBgn0031074 |
| *MED11* | -2.21677 | 0.174284 | FBgn0036811 |
| *CG13463* | -2.20749 | 0.179024 | FBgn0036470 |
| *Tsc1* | -2.20504 | 0.180303 | FBgn0026317 |
| *CG4841* | -2.20024 | 0.182379 | FBgn0032622 |
| *CG9581* | -2.19636 | 0.892665 | FBgn0031093 |
| *Nup153* | -2.19518 | 0.99905 | FBgn0061200 |
| *CG2909* | -2.1854 | 0.8023 | FBgn0030189 |
| *CG5787* | -2.18492 | 0.188863 | FBgn0032454 |
| *CG12546* | -2.18136 | 0.190228 | FBgn0037178 |
| *CG7766* | -2.15757 | 0.92772 | FBgn0030087 |
| *CG10137* | -2.15197 | 0.203386 | FBgn0032800 |
| *U2af50* | -2.15082 | 0.532692 | FBgn0005411 |
| *Su(var)205* | -2.14347 | 0.207438 | FBgn0003607 |
| *CG11617* | -2.12596 | 0.213827 | FBgn0031232 |
| *CG16947* | -2.12027 | 0.215673 | FBgn0031816 |
| *Sec61beta* | -2.1195 | 0.388059 | FBgn0010638 |
| *CG12654* | -2.11806 | 0.396059 | FBgn0030131 |
| *Uba1* | -2.10789 | 0.220246 | FBgn0023143 |
| *tacc* | -2.10118 | 0.530298 | FBgn0026620 |
| *ple* | -2.09431 | 0.227319 | FBgn0005626 |
| *CG14656* | -2.07022 | 0.241225 | FBgn0037278 |
| *eIF-5A* | -2.06579 | 0.242404 | FBgn0034967 |
| *CG32121* | -2.06359 | 0.242605 | FBgn0052121 |
| *Taf1* | -2.05763 | 0.245747 | FBgn0010355 |
| *acj6* | -2.05054 | 0.87058 | FBgn0000028 |
| *CG6181* | -2.04315 | 0.252573 | FBgn0032340 |
| *CG17472* | -2.03455 | 0.25651 | FBgn0032868 |
| *CG14218* | -2.03365 | 0.766847 | FBgn0031031 |
| *nudC* | -2.03317 | 0.257135 | FBgn0021768 |
| *alpha-Cat* | -2.02805 | 0.644805 | FBgn0010215 |
| *SoxN* | -2.01651 | 0.878511 | FBgn0029123 |
| *CG15731* | -2.01545 | 0.310031 | FBgn0030390 |
| *CG12934* | -2.01307 | 0.271427 | FBgn0033541 |
| *CG3726* | -2.01184 | 0.99905 | FBgn0029824 |
| *CG32202* | -2.01081 | 0.272339 | FBgn0052202 |
| *CG31313* | -2.00002 | 0.276051 | FBgn0051313 |
| *shi* | -1.99841 | 0.418714 | FBgn0003392 |
| *CG32704* | -1.97592 | 0.159405 | FBgn0052704 |
| *Osi14* | -1.96761 | 0.293742 | FBgn0040279 |
| *CG1688* | -1.96156 | 0.297623 | FBgn0027589 |
| *CG9617* | -1.9534 | 0.302506 | FBgn0037568 |
| *kst* | -1.95276 | 0.302585 | FBgn0004167 |
| *Scp1* | -1.94557 | 0.30521 | FBgn0020908 |
| *CG3770* | -1.94208 | 0.307075 | FBgn0035085 |
| *Txl* | -1.93943 | 0.307752 | FBgn0035631 |
| *Dat* | -1.91398 | 0.322404 | FBgn0019643 |
| *CG3277* | -1.91237 | 0.322593 | FBgn0031518 |
| *Rpp30* | -1.88692 | 0.336946 | FBgn0022246 |
| *Pde9* | -1.8443 | 0.449001 | FBgn0052648 |
| *tho2* | -1.84139 | 0.366702 | FBgn0031390 |
| *CG4959* | -1.84028 | 0.367033 | FBgn0028883 |
| *CG32112* | -1.83204 | 0.370552 | FBgn0052112 |
| *Pka-R1* | -1.81535 | 0.546005 | FBgn0000275 |
| *CG15458* | -1.79506 | 0.396079 | FBgn0040651 |
| *CG32056* | -1.79107 | 0.396431 | FBgn0052056 |
| *CG4615* | -1.75724 | 0.99905 | FBgn0029935 |
| *mus101* | -1.75279 | 0.398623 | FBgn0002878 |
| *CG16781* | -1.74324 | 0.423807 | FBgn0029661 |
