# Supplementary material for: COPI Complex Is a Regulator of Lipid Homeostasis
Source: PLoS Biol. 2008 Nov 25;6(11):e292. doi: 10.1371/journal.pbio.0060292 (PMC2586367; doi:10.1371/journal.pbio.0060292)
Supplement: Table S3 — (273 KB DOC) [file pbio.0060292.st003.doc]

**Table S3: Genes showing an overstorage phenotype in the primary *Drosophila* RNAi screen.**

Given are the *Drosophila* gene symbol, the average B-score normalized LD area per nuclei area value of the wells reaching the thresholding criteria (average B.Score) and the average Benjamini and Hochberg corrected p-value of the corresponding Amplicon(s) targeting the gene (note: a p-value greater than 0.05 might indicate a) additional, non-scoring wells or b) additional, non-scoring Amplicons targeting the given gene; B.Score.pVal). Additionally, the Flybase identifier (Flybase_id) is included.

| ***Drosophila* Gene Symbol** | **average B.Score** | **B.Score.pVal** | **Flybase_id** |
| --- | --- | --- | --- |
| *Gfat2* | 23.16731 | 4.23E-151 | FBgn0039580 |
| *zetaCOP* | 11.12168 | 3.58E-34 | FBgn0040512 |
| *CG9836* | 10.22147 | 1.05E-28 | FBgn0037637 |
| *RpL24* | 9.972007 | 2.75E-27 | FBgn0032518 |
| *eIF-2beta* | 9.616722 | 2.62E-25 | FBgn0004926 |
| *Fs(2)Ket* | 9.376763 | 5.12E-24 | FBgn0000986 |
| *CG7630* | 8.958292 | 7.45E-22 | FBgn0040793 |
| *RpL12* | 8.725786 | 1.10E-20 | FBgn0034968 |
| *Ctr1B* | 8.454395 | 2.25E-19 | FBgn0062412 |
| *RpL40* | 8.252179 | 2.04E-18 | FBgn0003941 |
| *RpS23* | 8.247617 | 2.07E-18 | FBgn0033912 |
| *CG3825* | 8.056219 | 1.57E-17 | FBgn0034948 |
| *CG6782* | 7.84242 | 1.48E-16 | FBgn0037912 |
| *brm* | 7.774321 | 2.89E-16 | FBgn0000212 |
| *CG4589* | 7.629874 | 1.24E-15 | FBgn0019886 |
| *slmo* | 7.452184 | 7.25E-15 | FBgn0029161 |
| *CG7263* | 7.405874 | 1.12E-14 | FBgn0031392 |
| *CG14542* | 7.219804 | 6.57E-14 | FBgn0039402 |
| *l(2)NC136* | 7.053397 | 2.98E-13 | FBgn0033029 |
| *alphaCop* | 6.927919 | 9.44E-13 | FBgn0025725 |
| *a* | 6.758184 | 4.18E-12 | FBgn0000008 |
| *smi21F* | 6.701131 | 6.70E-12 | FBgn0016926 |
| *Qm* | 6.596014 | 1.67E-11 | FBgn0024733 |
| *deltaCOP* | 6.57421 | 2.01E-11 | FBgn0028969 |
| *CG32809* | 6.44037 | 0.000113 | FBgn0023531 |
| *CG14755* | 6.383551 | 9.80E-11 | FBgn0033282 |
| *RpL21* | 6.257513 | 2.83E-10 | FBgn0032987 |
| *CG7580* | 6.244581 | 3.04E-10 | FBgn0036728 |
| *eIF3-S10* | 6.083208 | 1.14E-09 | FBgn0037249 |
| *CG7181* | 6.066507 | 1.28E-09 | FBgn0037097 |
| *Pabp2* | 6.006319 | 2.06E-09 | FBgn0005648 |
| *Hsc70-1* | 5.958778 | 2.89E-09 | FBgn0001216 |
| *gammaCop* | 5.939696 | 3.29E-09 | FBgn0028968 |
| *eIF4G* | 5.860523 | 5.95E-09 | FBgn0023213 |
| *RpL30* | 5.826655 | 7.49E-09 | FBgn0015745 |
| *CG9053* | 5.785882 | 9.38E-09 | FBgn0030606 |
| *RpL11* | 5.678217 | 2.05E-08 | FBgn0013325 |
| *CG30387* | 5.676523 | 2.05E-08 | FBgn0050387 |
| *CG5151* | 5.673196 | 2.07E-08 | FBgn0036576 |
| *CG13445* | 5.653474 | 2.37E-08 | FBgn0036532 |
| *CG12170* | 5.597008 | 0.00022 | FBgn0037356 |
| *CG31666* | 5.591519 | 3.57E-08 | FBgn0051666 |
| *CG13623* | 5.585417 | 3.68E-08 | FBgn0039205 |
| *Dr* | 5.377036 | 0.023381 | FBgn0000492 |
| *CG5953* | 5.360166 | 1.72E-07 | FBgn0032587 |
| *Hsc70-3* | 5.353329 | 2.55E-11 | FBgn0001218 |
| *RpL9* | 5.338987 | 1.98E-07 | FBgn0015756 |
| *CG9603* | 5.315584 | 2.32E-07 | FBgn0040529 |
| *Arf79F* | 5.313745 | 3.30E-06 | FBgn0010348 |
| *CG8179* | 5.297027 | 0.000135 | FBgn0034020 |
| *RpL18* | 5.262522 | 3.36E-07 | FBgn0035753 |
| *RpL23* | 5.25555 | 3.48E-07 | FBgn0010078 |
| *CG2147* | 5.253863 | 0.001279 | FBgn0030025 |
| *beta'Cop* | 5.10747 | 9.36E-07 | FBgn0025724 |
| *CG1884* | 5.088685 | 1.03E-06 | FBgn0033424 |
| *CG33128* | 5.03226 | 0.026297 | FBgn0053128 |
| *Cyt-c-p* | 4.987484 | 1.95E-06 | FBgn0000409 |
| *CG17331* | 4.96484 | 2.20E-06 | FBgn0032596 |
| *RpL14* | 4.926606 | 2.78E-06 | FBgn0017579 |
| *thr* | 4.906561 | 3.15E-06 | FBgn0003701 |
| *RpL17* | 4.886449 | 0.005737 | FBgn0029897 |
| *mtrm* | 4.860122 | 4.19E-06 | FBgn0010431 |
| *MED6* | 4.84124 | 4.70E-06 | FBgn0024330 |
| *CG9140* | 4.830851 | 4.95E-06 | FBgn0031771 |
| *His-Psi:CR31754* | 4.762034 | 7.47E-06 | FBgn0051754 |
| *CG5794* | 4.754248 | 7.79E-06 | FBgn0039214 |
| *Rho1* | 4.725214 | 0.33718 | FBgn0014020 |
| *ND75* | 4.696833 | 1.09E-05 | FBgn0017566 |
| *CG13504* | 4.685555 | 1.16E-05 | FBgn0034699 |
| *mRpS18A* | 4.666765 | 1.29E-05 | FBgn0051450 |
| *RpS29* | 4.652528 | 1.40E-05 | FBgn0037752 |
| *CG10664* | 4.636935 | 1.53E-05 | FBgn0032833 |
| *mRpL51* | 4.626295 | 1.63E-05 | FBgn0032053 |
| *betaCop* | 4.62465 | 1.63E-05 | FBgn0008635 |
| *CG9240* | 4.599103 | 1.89E-05 | FBgn0030669 |
| *RpL39* | 4.580279 | 2.11E-05 | FBgn0023170 |
| *RpL31* | 4.567905 | 2.26E-05 | FBgn0025286 |
| *RpL10Ab* | 4.542755 | 2.57E-05 | FBgn0036213 |
| *RpL38* | 4.525519 | 2.79E-05 | FBgn0040007 |
| *CG17493* | 4.514786 | 2.96E-05 | FBgn0040010 |
| *Tbp* | 4.504812 | 3.13E-05 | FBgn0003687 |
| *CG7177* | 4.46356 | 3.95E-05 | FBgn0037098 |
| *pAbp* | 4.455146 | 4.13E-05 | FBgn0003031 |
| *Hsp70Ba* | 4.422884 | 4.96E-05 | FBgn0013277, FBgn0013278, FBgn0013275, FBgn0013279, FBgn0013276, FBgn0051354 |
| *Lim3* | 4.405991 | 0.012134 | FBgn0002023 |
| *Pk61C* | 4.405842 | 5.47E-05 | FBgn0020386 |
| *Hs3st-A* | 4.364507 | 6.81E-05 | FBgn0053147 |
| *tin* | 4.347706 | 7.50E-05 | FBgn0004110 |
| *CG6330* | 4.340525 | 7.77E-05 | FBgn0039464 |
| *CG40337* | 4.313864 | 8.98E-05 | FBgn0058337 |
| *CG14115* | 4.304039 | 9.36E-05 | FBgn0036343 |
| *CG5385* | 4.303753 | 9.36E-05 | FBgn0032215 |
| *Trip1* | 4.303425 | 9.36E-05 | FBgn0015834 |
| *CG31453* | 4.296849 | 9.61E-05 | FBgn0051453 |
| *geminin* | 4.278097 | 0.000105 | FBgn0033081 |
| *CG32720* | 4.266409 | 0.000112 | FBgn0052720 |
| *Tango1* | 4.256115 | 0.000118 | FBgn0031842 |
| *CG15157* | 4.255192 | 0.000176 | FBgn0032675 |
| *Krn* | 4.229272 | 0.000134 | FBgn0052179 |
| *bru-2* | 4.204906 | 0.000151 | FBgn0043904 |
| *bmm* | 4.176621 | 9.64E-07 | FBgn0036449 |
| *D19A* | 4.145512 | 0.000205 | FBgn0022935 |
| *RpS11* | 4.135058 | 0.053112 | FBgn0033699 |
| *Su(var)3-9* | 4.109441 | 0.000243 | FBgn0003600 |
| *l(1)G0136* | 4.061435 | 0.000312 | FBgn0026666 |
| *RpL4* | 4.050351 | 0.00033 | FBgn0003279 |
| *CG6179* | 4.045412 | 0.000336 | FBgn0030915 |
| *Ctr1A* | 4.038834 | 0.000346 | FBgn0062413 |
| *CG16791* | 4.035522 | 0.000351 | FBgn0038881 |
| *CG4169* | 4.015319 | 0.000385 | FBgn0036642 |
| *CG8043* | 4.012617 | 0.000389 | FBgn0037610 |
| *RpS4* | 4.009864 | 0.000393 | FBgn0011284 |
| *CG32733* | 4.007924 | 2.54E-08 | FBgn0052733, FBgn0052797 |
| *RpL19* | 4.006941 | 0.000397 | FBgn0002607 |
| *CG3213* | 3.991668 | 0.274004 | FBgn0031545 |
| *dpr17* | 3.982203 | 0.000451 | FBgn0051361 |
| *RpL7* | 3.970279 | 0.000477 | FBgn0005593 |
| *CG7185* | 3.969412 | 0.000477 | FBgn0035872 |
| *RpLP0* | 3.967129 | 0.000481 | FBgn0000100 |
| *rnh1* | 3.964503 | 0.000483 | FBgn0023171 |
| *Best3* | 3.929281 | 0.000574 | FBgn0036492 |
| *CG6770* | 3.928194 | 0.090835 | FBgn0032400 |
| *CG5844* | 3.896785 | 0.000679 | FBgn0038049 |
| *mRpL39* | 3.893395 | 0.000688 | FBgn0036462 |
| *CG4644* | 3.866415 | 0.000781 | FBgn0031300 |
| *Hsp70Ba* | 3.858672 | 0.000806 | FBgn0013275, FBgn0013276 |
| *RpL18A* | 3.857169 | 0.000807 | FBgn0010409 |
| *Tektin-C* | 3.855951 | 0.000807 | FBgn0035638 |
| *eIF-4a* | 3.841633 | 0.000868 | FBgn0001942 |
| *ths* | 3.837265 | 0.000885 | FBgn0033652 |
| *CG14030* | 3.826481 | 0.000932 | FBgn0031696 |
| *RpL27* | 3.814354 | 0.000985 | FBgn0039359 |
| *CG13716* | 3.784588 | 0.001138 | FBgn0035563 |
| *CG9007* | 3.770962 | 0.001217 | FBgn0036398 |
| *Nup358* | 3.722511 | 0.001497 | FBgn0039302 |
| *Hsp70Bb* | 3.706255 | 0.025077 | FBgn0013277, FBgn0013278, FBgn0013279, FBgn0051354 |
| *Snap* | 3.688584 | 0.010203 | FBgn0011712 |
| *mRpL54* | 3.687676 | 0.001776 | FBgn0034579 |
| *CG15489* | 3.681781 | 0.001823 | FBgn0032438 |
| *CG31554* | 3.632933 | 0.002274 | FBgn0051554 |
| *rin* | 3.63083 | 0.002283 | FBgn0015778 |
| *CG9216* | 3.623418 | 0.004268 | FBgn0030726 |
| *RpLP1* | 3.619587 | 0.002395 | FBgn0002593 |
| *Cirl* | 3.613162 | 0.002466 | FBgn0033313 |
| *CG9865* | 3.609594 | 0.00249 | FBgn0034649 |
| *CG13743* | 3.60239 | 0.002562 | FBgn0033368 |
| *CG7172* | 3.593958 | 0.002663 | FBgn0037102 |
| *CG30336* | 3.579126 | 0.002837 | FBgn0050336 |
| *DnaJ-60* | 3.565306 | 0.003015 | FBgn0020129 |
| *CG6873* | 3.557744 | 0.003119 | FBgn0030951 |
| *RpS6* | 3.549274 | 0.014439 | FBgn0004922 |
| *sesB* | 3.549094 | 0.010619 | FBgn0003360 |
| *CG7597* | 3.548582 | 0.003611 | FBgn0037093 |
| *Ac78C* | 3.548324 | 0.003244 | FBgn0024150 |
| *CG14543* | 3.519243 | 0.003693 | FBgn0039404 |
| *zormin* | 3.517835 | 0.003703 | FBgn0052311 |
| *RpL10Aa* | 3.515744 | 0.003728 | FBgn0038281 |
| *l(2)k14708* | 3.505634 | 0.003862 | FBgn0021848 |
| *how* | 3.480665 | 0.004302 | FBgn0017397 |
| *Int6* | 3.475339 | 0.004384 | FBgn0025582 |
| *Samuel* | 3.455648 | 0.004765 | FBgn0032330 |
| *CG12904* | 3.452521 | 0.004813 | FBgn0033510 |
| *trpl* | 3.448161 | 0.239364 | FBgn0005614 |
| *RpS19a* | 3.436156 | 0.005194 | FBgn0010412 |
| *RpL13* | 3.42534 | 0.005432 | FBgn0011272 |
| *CG3760* | 3.404652 | 0.005897 | FBgn0022343 |
| *grau* | 3.401434 | 0.005959 | FBgn0001133 |
| *CG31302* | 3.350593 | 0.007329 | FBgn0051302 |
| *CG32773* | 3.349717 | 0.007329 | FBgn0052773 |
| *CG33256* | 3.34915 | 0.007329 | FBgn0053256 |
| *CG32193* | 3.346088 | 0.007413 | FBgn0052193 |
| *CG5674* | 3.326111 | 0.008037 | FBgn0032656 |
| *CG30290* | 3.302079 | 0.172097 | FBgn0050290 |
| *blw* | 3.297001 | 0.009118 | FBgn0011211 |
| *CG6950* | 3.272084 | 0.01014 | FBgn0037955 |
| *Pdp1* | 3.267162 | 0.010306 | FBgn0016694 |
| *CG10713* | 3.259397 | 0.010616 | FBgn0036360 |
| *eIF3-S8* | 3.233874 | 0.011507 | FBgn0034258 |
| *Tango7* | 3.223868 | 0.011973 | FBgn0033902 |
| *CG4186* | 3.221598 | 0.012008 | FBgn0040634 |
| *CHES-1-like* | 3.21687 | 0.012152 | FBgn0029504 |
| *RpS20* | 3.210344 | 0.012479 | FBgn0019936 |
| *CG6852* | 3.199967 | 0.013042 | FBgn0036820 |
| *CG5213* | 3.184844 | 0.013802 | FBgn0038345 |
| *CG9769* | 3.168923 | 0.014696 | FBgn0037270 |
| *CG10660* | 3.159734 | 0.015157 | FBgn0036288 |
| *bip2* | 3.157166 | 0.015275 | FBgn0026262 |
| *RpS24* | 3.131259 | 0.976114 | FBgn0034751 |
| *CG7039* | 3.125255 | 0.042407 | FBgn0030088 |
| *RpII215* | 3.112925 | 0.665043 | FBgn0003277 |
| *CG12022* | 3.11284 | 0.017996 | FBgn0035276 |
| *lola* | 3.111609 | 0.018045 | FBgn0005630 |
| *stich1* | 3.109678 | 0.018053 | FBgn0016941 |
| *Dcp2* | 3.101557 | 0.009117 | FBgn0036534 |
| *CG2574* | 3.080933 | 0.061447 | FBgn0030386 |
| *CG6188* | 3.060979 | 0.021419 | FBgn0038074 |
| *Mes2* | 3.045486 | 0.847932 | FBgn0037207 |
| *Fas3* | 3.041118 | 0.023003 | FBgn0000636 |
| *Dbp45A* | 3.040066 | 0.023046 | FBgn0010220 |
| *cana* | 3.033583 | 0.023406 | FBgn0040233 |
| *Nacalpha* | 3.017284 | 0.024857 | FBgn0017565 |
| *CG31847* | 3.016018 | 0.024873 | FBgn0051847 |
| *Tis11* | 2.99196 | 0.054567 | FBgn0011837 |
| *CG7918* | 2.967212 | 0.029459 | FBgn0037546 |
| *disp* | 2.959913 | 0.030239 | FBgn0029088 |
| *mRpL18* | 2.951515 | 0.031226 | FBgn0026741 |
| *CR33319* | 2.949746 | 0.03134 | FBgn0053319 |
| *CG6404* | 2.946703 | 0.031674 | FBgn0027615 |
| *CG32105* | 2.936307 | 0.032778 | FBgn0052105 |
| *CG5532* | 2.933455 | 0.033018 | FBgn0034902 |
| *CG15133* | 2.929096 | 0.033473 | FBgn0032619 |
| *CG33274* | 2.924981 | 0.033823 | FBgn0035864 |
| *RpL27A* | 2.91708 | 0.034565 | FBgn0010410 |
| *CG8960* | 2.907164 | 0.035463 | FBgn0035315 |
| *bgm* | 2.895454 | 0.037066 | FBgn0027348 |
| *RpL6* | 2.89205 | 0.037505 | FBgn0039857 |
| *CG13235* | 2.885416 | 0.03844 | FBgn0040766 |
| *CG6094* | 2.885122 | 0.597671 | FBgn0032261 |
| *RpL26* | 2.875088 | 0.039696 | FBgn0036825 |
| *His4r* | 2.865847 | 0.040879 | FBgn0013981 |
| *MrgBP* | 2.8501 | 0.042894 | FBgn0033341 |
| *His3.3A* | 2.843538 | 0.043745 | FBgn0014857 |
| *CG14107* | 2.83939 | 0.044396 | FBgn0036351 |
| *CG33271* | 2.829487 | 0.045783 | FBgn0053271 |
| *CG11453* | 2.829263 | 0.045783 | FBgn0038734 |
| *CG7549* | 2.816055 | 0.047612 | FBgn0037559 |
| *His3:CG31613* | 2.809515 | 0.048675 | FBgn0051613 |
| *SK* | 2.80024 | 0.05015 | FBgn0029761 |
| *dpr8* | 2.794888 | 0.050799 | FBgn0052600 |
| *CG14304* | 2.790713 | 0.051143 | FBgn0038629 |
| *mRpL15* | 2.77439 | 0.151055 | FBgn0036990 |
| *CG3618* | 2.76917 | 0.481737 | FBgn0037028 |
| *CG12236* | 2.764767 | 0.636384 | FBgn0029822 |
| *l(1)G0060* | 2.76144 | 0.034846 | FBgn0027321 |
| *CG11208* | 2.759352 | 0.05578 | FBgn0034488 |
| *CG11048* | 2.750517 | 0.0574 | FBgn0034487 |
| *RpL36A* | 2.73557 | 0.059644 | FBgn0031980 |
| *CG14721* | 2.734082 | 0.059774 | FBgn0037942, FBgn0002906 |
| *mRpL2* | 2.726283 | 0.061233 | FBgn0036135 |
| *pxt* | 2.723871 | 0.061427 | FBgn0038538 |
| *l(3)03670* | 2.720994 | 0.061785 | FBgn0010808 |
| *ATPsyn-d* | 2.71962 | 0.062005 | FBgn0016120 |
| *CG31284* | 2.691465 | 0.567691 | FBgn0051284 |
| *mRpL45* | 2.669409 | 0.072837 | FBgn0038996 |
| *Mys45A* | 2.668904 | 0.072854 | FBgn0033379 |
| *CG31158* | 2.664061 | 0.074092 | FBgn0051158 |
| *RpS3A* | 2.654511 | 0.076327 | FBgn0017545 |
| *CG3363* | 2.647003 | 0.078278 | FBgn0034987 |
| *DNApol-alpha180* | 2.639449 | 0.079877 | FBgn0004493 |
| *CG4825* | 2.636746 | 0.080566 | FBgn0037010 |
| *CG9757* | 2.631787 | 0.081409 | FBgn0003060 |
| *Argk* | 2.62865 | 0.081652 | FBgn0000116 |
| *Chrac-16* | 2.626004 | 0.227319 | FBgn0043001 |
| *Gtp-bp* | 2.62514 | 0.039875 | FBgn0010391 |
| *Arp87C* | 2.614605 | 0.085438 | FBgn0011745 |
| *CG31291* | 2.607566 | 0.122766 | FBgn0051291 |
| *RpL3* | 2.597142 | 0.090233 | FBgn0020910 |
| *CG8509* | 2.58946 | 0.733464 | FBgn0030696 |
| *Osi19* | 2.585585 | 0.093426 | FBgn0037429 |
| *CG9065* | 2.572422 | 0.097342 | FBgn0030610 |
| *CG32219* | 2.570684 | 0.097815 | FBgn0052219 |
| *RpS5a* | 2.567463 | 0.098674 | FBgn0002590 |
| *fau* | 2.553233 | 0.102705 | FBgn0020439 |
| *CG2577* | 2.548949 | 0.202729 | FBgn0030384 |
| *RpL22* | 2.539883 | 0.106541 | FBgn0015288 |
| *CoVa* | 2.537836 | 0.107015 | FBgn0019624 |
| *RpS27A* | 2.52909 | 0.109315 | FBgn0003942 |
| *Atpalpha* | 2.526125 | 0.110075 | FBgn0002921 |
| *CG13300* | 2.525941 | 0.110075 | FBgn0035699 |
| *mRpS9* | 2.517697 | 0.473337 | FBgn0037529 |
| *Osi8* | 2.515784 | 0.113546 | FBgn0037415 |
| *hoip* | 2.504847 | 0.116739 | FBgn0015393 |
| *PGRP-LC* | 2.494745 | 0.120743 | FBgn0035976 |
| *AcCoAS* | 2.491432 | 0.121468 | FBgn0012034 |
| *cno* | 2.488717 | 0.122488 | FBgn0000340 |
| *l(2)06225* | 2.487457 | 0.648453 | FBgn0010612 |
| *CkIalpha* | 2.444635 | 0.35931 | FBgn0015024 |
| *CG15888* | 2.443482 | 0.139111 | FBgn0038131 |
| *beat-VII* | 2.440334 | 0.139388 | FBgn0039447 |
| *CG1545* | 2.440156 | 0.139388 | FBgn0030259 |
| *CG13216* | 2.440101 | 0.139388 | FBgn0033591 |
| *CG1874* | 2.436736 | 0.139773 | FBgn0033425 |
| *tsr* | 2.435621 | 0.139773 | FBgn0011726 |
| *repo* | 2.435613 | 0.139773 | FBgn0011701 |
| *Nc73EF* | 2.434413 | 0.139773 | FBgn0010352 |
| *CG15891* | 2.433484 | 0.99905 | FBgn0029860 |
| *NetB* | 2.42008 | 0.560439 | FBgn0015774 |
| *Hsp70Bc* | 2.415228 | 0.1476 | FBgn0013279 |
| *CG30007* | 2.41241 | 0.148596 | FBgn0050007 |
| *fred* | 2.410293 | 0.149158 | FBgn0051774 |
| *CG4695* | 2.406432 | 0.150196 | FBgn0037859 |
| *CG13739* | 2.403986 | 0.151055 | FBgn0033403 |
| *Gr98a* | 2.392163 | 0.155755 | FBgn0039520 |
| *DMAP1* | 2.364024 | 0.165549 | FBgn0034537 |
| *Hsc70-2* | 2.358413 | 0.167707 | FBgn0001217 |
| *mRpS34* | 2.350696 | 0.170263 | FBgn0036613 |
| *CG9392* | 2.347714 | 0.171148 | FBgn0036895 |
| *CG7556* | 2.33388 | 0.177234 | FBgn0030990 |
| *CG13717* | 2.317228 | 0.184835 | FBgn0035562 |
| *CG15772* | 2.315243 | 0.185157 | FBgn0029799 |
| *raw* | 2.314735 | 0.185157 | FBgn0003209 |
| *CG1967* | 2.313638 | 0.322593 | FBgn0030341 |
| *CG32050* | 2.307081 | 0.188996 | FBgn0052050 |
| *CG15312* | 2.288124 | 0.953053 | FBgn0030174 |
| *mRpS30* | 2.284689 | 0.404531 | FBgn0030692 |
| *MED25* | 2.281476 | 0.200497 | FBgn0038760 |
| *CG32633* | 2.267977 | 0.207043 | FBgn0052633 |
| *CG13942* | 2.265664 | 0.207438 | FBgn0033922 |
| *mRpL1* | 2.244763 | 0.214872 | FBgn0037566 |
| *CG15908* | 2.235148 | 0.218413 | FBgn0033085 |
| *RpL8* | 2.178661 | 0.246129 | FBgn0024939 |
| *CG33308* | 2.138577 | 0.269608 | FBgn0053308 |
| *mTTF* | 2.134549 | 0.272004 | FBgn0028530 |
| *CR15280* | 2.125735 | 0.275824 | FBgn0028867 |
| *RpL28* | 2.125029 | 0.275824 | FBgn0035422 |
| *CG32778* | 2.109499 | 0.28266 | FBgn0052778 |
| *CG33300* | 2.092058 | 0.481199 | FBgn0053300 |
| *CG14306* | 2.080895 | 0.299455 | FBgn0038628 |
